# Invasive hemodynamic monitoring-guided resuscitation improves survival in shock: A systematic review and meta-analysis

**DOI:** 10.1016/j.aicoj.2026.100071

**Published:** 2026-04-29

**Authors:** Liliána Nagy, Petra Réka Tóth, Caner Turan, Dávid Laczkó, Lili Légár, Basak Danisan, Zoltán Sipos, Péter Hegyi, Zsolt Molnár, László Zubek

**Affiliations:** a Centre for Translational Medicine, Semmelweis University, Budapest, Hungary; b Department of Anesthesiology and Intensive Therapy, Semmelweis University, Budapest, Hungary; c Department of Interventional Radiology, Heart and Vascular Centre, Semmelweis University, Budapest, Hungary; d Institute for Translational Medicine, Medical School, University of Pécs, Pécs, Hungary; e Institute of Pancreatic Diseases, Semmelweis University, Budapest, Hungary; f Department of Anaesthesiology and Intensive Therapy, Poznan University of Medical Sciences, Faculty of Medicine, Poznan, Poland

**Keywords:** Shock, Instability, Hemodynamic monitoring, Pulmonary artery catheter, Swan-Ganz catheter, Thermodilution, PiCCO

## Abstract

**Background:**

Mortality in shock may reach 60%; hence, immediate, adequate resuscitation has a crucial role in improving outcomes. Detailed hemodynamic monitoring is desirable, but evidence on its outcome benefits is limited. Therefore, this study aimed to compare advanced hemodynamic monitoring (AHDM)-guided clinical decision-making and treatment with conventional ones in terms of outcomes in shock.

**Methods:**

A systematic search was performed in three databases (PubMed, EMBASE, and Cochrane Library) until 9 November 2024. Randomised controlled trials, non-randomised and observational studies involving adult shock patients were eligible for inclusion. Main outcomes were in-hospital and 30-day mortality, and secondary outcomes included length of stay, need for and duration of organ support, and amount of fluid administered. Meta-analyses were performed using a random-effects model, with heterogeneity and risk of bias assessed. The review protocol was registered in PROSPERO (ID: CRD42024607758).

**Results:**

A total of 34 studies, including seven RCTs and 636,441 shock patients, were analysed to compare AHDM with conventional monitoring. The use of any type of AHDM was associated with a significantly lower in-hospital mortality for any type of shock (OR: 0.66; 95% CI: [0.48; 0.91], *p* = 0.014), especially in cardiogenic shock patients, managed with pulmonary artery catheter-guided therapy (OR: 0.68, 95% CI: [0.60; 0.78], *p* < 0.001). This contrasts with the significantly higher odds of requiring organ support treatment, including inotropes (OR: 2.32, CI: [1.29; 4.19], *p* = 0.012), vasopressors (OR: 1.46, CI: [1.05; 2.04], *p* = 0.030), mechanical circulatory support (OR: 2.85, CI: [1.62; 5.02], *p* = 0.002), renal replacement therapy or mechanical ventilation, although the duration of mechanical ventilation was shorter. Heterogeneity was predictably high due to the variety of shock types and monitoring methods. Risk of bias was predominantly low in RCTs and serious in observational studies.

**Conclusion:**

AHDM use is associated with a significant reduction in mortality in shock patients, with the greatest benefit observed in cardiogenic shock. The observed outcomes suggest that AHDM may facilitate qualitative changes in decision-making, consistent with precision-guided resuscitation.

## Introduction

Shock continues to be a major cause of morbidity and mortality in critically ill patients, with mortality rates ranging from 30% to 60% depending on etiology and severity [1,2]. The main determinant of early death is hemodynamic instability [3], yet protocols for hemodynamic monitoring and resuscitation are based on limited evidence [4,5].

Conventional monitoring is widely available but often unreliable in identifying inadequate tissue perfusion [6]. Advanced hemodynamic monitoring (AHDM) systems, such as the pulmonary artery catheter (PAC), Pulse index Continuous Cardiac Output (PiCCO), and Lithium Dilution Cardiac Output (LiDCO), allow direct measurement of cardiac output and derived indices, offering a more comprehensive assessment of circulatory status. PAC is generally reserved for patients with severe or refractory hemodynamic instability [[7], [8], [9]]. Modern critical care increasingly favours less invasive technologies, such as PiCCO and LiDCO, reflecting a shift towards minimising procedural risk. However, accuracy and reliability remain essential, as therapeutic decisions based on imprecise measurements may compromise patient safety [10]. Conventional invasive or non-calibrated monitoring provides limited hemodynamic information. AHDM can offer more detailed, accessible and repeatable measurements to guide clinical decision-making, although its effective use requires both technical expertise for device insertion, incorporation of dynamic assessments, and careful interpretation of hemodynamic data within the comprehensive clinical picture. Echocardiography is a crucial tool for assessing cardiac structure and function, but it is more time- and operator-dependent. Despite these advances, evidence that AHDM improves patient-centred outcomes remains inconclusive. Some trials and cohort studies suggest improved hemodynamic control and patient outcomes [11,12], whereas others show a neutral effect on survival and higher complication rates [13,14]. Due to these inconsistencies, guidelines acknowledge the potential of AHDM in complex shock but lack evidence to recommend its use, leaving clinicians without clear guidance [4,5].

Given this uncertainty, a comprehensive synthesis of the available evidence is needed to determine whether AHDM contributes to better patient outcomes. We therefore conducted a systematic review and meta-analysis to evaluate whether AHDM-guided therapy, using devices such as PAC, PiCCO, or LiDCO, improves outcomes compared with conventional hemodynamic management in adult patients with shock. Our findings aim to support evidence-based decision-making and help define the appropriate role of AHDM in shock management.

## Methods

We conducted and reported this systematic review and meta-analysis in accordance with the PRISMA 2020 guidelines [15] and followed the methodological recommendations of the Cochrane Handbook for Systematic Reviews of Interventions. The study protocol was registered on PROSPERO (registration number: CRD42024607758), and the review was conducted in accordance with it. We deviated slightly from our registered protocol in terms of the analysis of organ support-free days: as the authors reported the duration of organ support rather than the organ support-free time, we focused our analysis on the former outcome. This work was performed as part of the Systems Education Program at Semmelweis University [16] and conducted within the Translational Medicine (TM) Cycle Framework of the Academia Europaea [17].

### Eligibility criteria

Clinical trials, regardless of randomisation, retrospective and prospective observational studies were eligible for inclusion. Eligibility criteria were defined according to our PICO framework. The population (P) included adult patients (≥18 years) with shock of any etiology, or patients with hemodynamic instability or prolonged need for circulatory support. Populations including pregnant patients or those already on organ support were also eligible for inclusion. The intervention (I) was clinical decision-making and therapy guided by AHDM using PAC, PiCCO or LiDCO devices, or therapy based on parameters such as cardiac output, PCWP, EVLW, GEDV or ITBV. The comparator (C) was a conventional decision-making and treatment without AHDM. The primary outcome (O) was mortality (including both in-hospital and 30-day endpoints). Additional outcomes included the need for organ support, duration of organ support, length of hospital- and ICU stay, fluid volumes administered, organ dysfunction parameters (SOFA and MODS scores), and intervention-related complications.

We excluded case reports and case series, reviews, meta-analyses, abstracts, conference papers, letters, ongoing trials, and studies without full text. Pediatric and animal studies, and those including only hemodynamically stable patients, were not eligible. We also excluded studies using only one monitoring method without comparison, simultaneous use of multiple devices, non-invasive or uncalibrated devices (e.g., FloTrac, Vigileo), or comparison of two advanced methods without a conventional group. Studies with statistically incomparable results were also excluded.

### Information sources, search strategy

A systematic search was conducted in three electronic databases (PubMed Central, Cochrane Library, and Embase) on 9 November 2024, using a structured search key. The first domain addressed shock of various etiologies, and the second domain focused on devices (PAC, PiCCO, and LiDCO). No filters were applied for language or publication date. The detailed search key is provided in the supplementary material (Supplementary Material 1).

References of eligible articles were also examined using CitationChaser to identify further studies.

### Selection process

The selection process involved duplicate removal, followed by screening of titles and abstracts, and then full-text assessment for final inclusion. Two independent teams, each consisting of two reviewers (Team 1: LN and BD, Team 2: PRT and LL), conducted the study selection.

Agreement between reviewers was evaluated using Cohen’s kappa statistic, with values above 0.8 considered acceptable. Agreement between reviewers was assessed using Cohen’s kappa statistic: κ = 0.6 during title and abstract screening (discrepancies were not due to theoretical or methodological errors, but rather to ambiguities in the abstracts as to whether monitoring was applied to subgroups or all participants), and 0.9 during full-text review. Discrepancies were resolved through consensus-building meetings.

### Data collection process

Data from eligible articles were independently extracted by two teams, each consisting of two reviewers (Team 1: LN and LL, Team 2: PRT and BD), using the same standardised data extraction table (Supplementary Tables 2–4), developed with methodological and clinical experts to ensure consistency and relevance. Any discrepancies were addressed and resolved during a consensus meeting.

### Data items

The following data were extracted from each study: first author, year of publication, DOI, study design, study period, total number of patients, type of shock and the hemodynamic monitoring devices used in both the intervention and control groups. Group-specific data included the number of patients, gender distribution, age, BMI and comorbidities. Outcome data included in-hospital and 30-day mortality, as well as the number of patients requiring various types of organ support: non-invasive and mechanical ventilation, inotropic and vasopressor therapy, renal replacement therapy, and mechanical circulatory support (including intra-aortic balloon pump [IABP] and ventricular assist devices). Additional data were collected on the duration of mechanical ventilation, length of ICU and hospital stay, fluid volumes administered at various time points, complications and organ dysfunction scores.

### Study risk of bias assessment

Risk of bias was evaluated separately for each included study and each outcome by two teams of researchers (Team 1: LN and LL, Team 2: PRT and BD), using the RoB 2 tool for randomised trials and the ROBINS-I tool for non-randomised studies. In cases of disagreement, a structured resolution process was used to reach consensus. The certainty of evidence for all outcomes was evaluated using the GRADE approach [18,19].

### Synthesis methods

The meta-analysis was performed for outcomes for which data were reported in at least three studies to ensure robustness and reliability. Statistical analyses were conducted using R (R Core Team 2021, v4.1.1). Dichotomous outcomes are reported as odds ratios (OR) with 95% confidence intervals, while continuous outcomes are expressed as mean differences (MD) with standard deviations (SD). If the mean and SD were not reported in the article, they were estimated from the reported values of medians, quartiles and ranges. Forest plots were generated to calculate and illustrate each study outcome, using the META (Schwarzer 2022, v5.2.0) and DMETAR (Cuijpers, Furukawa, and Ebert 2022, v0.0.9000) packages. Statistical significance was set at *p* < 0.05 for all analyses. Based on our assumption of considerable between-study heterogeneity, a random-effects model was used to pool effect sizes in all cases. Heterogeneity was assessed by means using the means of the Cochrane Q (χ²) test and the I² statistic, and was considered statistically significant at *p* < 0.1. Publication bias was assessed using visual analysis of funnel plots and Egger's linear regression test for funnel plot asymmetry to screen for small-study effects [20].

Pre-specified subgroup analyses were conducted by shock etiology and study design. Device type was additionally defined a priori as a subgroup; based on data availability and interpretability, stratified analyses were conducted for mortality and fluid volume outcomes.

For the analysis of in-hospital mortality studies that did not specify the time frame of mortality were also included, considering that follow-up likely ended at hospital discharge. For 30-day mortality, studies reporting 28- and 30-day mortality data were pooled together.

For propensity score-matched observational studies, analyses were based on matched cohorts where available; unmatched data were used only when matched results were not reported.

When weighted data were analysed, effect estimates derived from propensity score-based inverse probability of treatment weighting were used to balance baseline patient and hospital characteristics, enabling adjusted comparisons between groups [21].

## Results

### Study selection

**Fig. 1:**
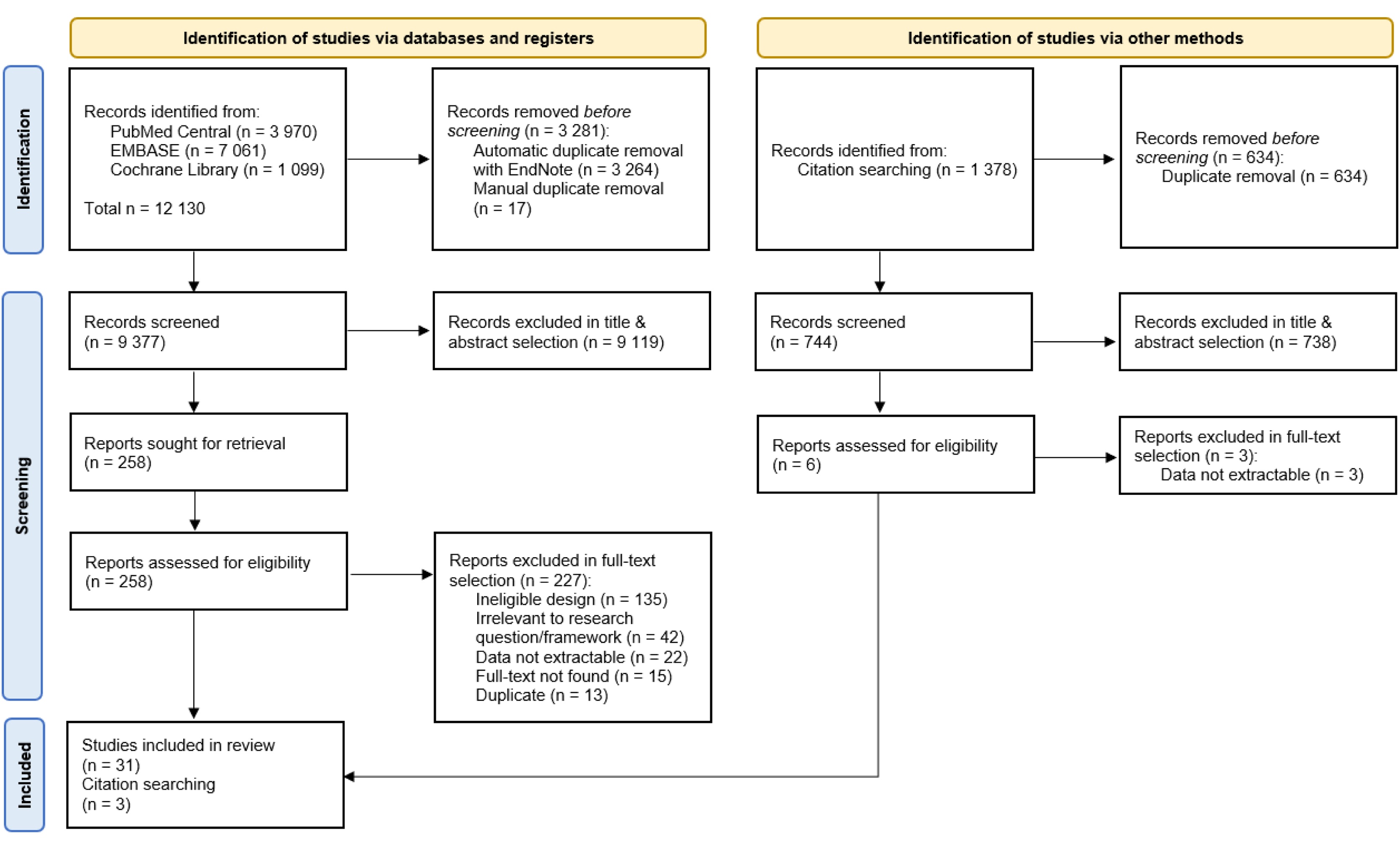
Study selection screening process.

The systematic search resulted in 12,130 identified records; after duplicate removal, 9,377 articles were screened by title and abstract, and 258 were selected for full-text review. A total of 34 studies were included in the final analysis, of which 31 studies were identified through the systematic search and three articles by citation chasing. The main reasons for exclusion were ineligible study design, lack of alignment with the research framework, and non-extractable data (authors were contacted when possible). Examples include outcomes reported outside the predefined timeframe, use of an unspecified AHDM device, or the absence of shock-specific data. The screening process is illustrated in Fig. 1.

### Study characteristics

The 34 eligible articles included seven RCTs, three non-randomised studies, and 24 observational studies (eight prospective, 16 retrospective), encompassing a total of 636,441 patients. Of these, 1,312 had septic, 632,971 cardiogenic, 78 trauma-related, and 2,080 mixed shock (either unspecified etiology or multiple shock types analysed together), with 100,286 patients receiving AHDM. Table 1 presents the most relevant baseline characteristics and specifies which studies reported each outcome, while the detailed version is provided in the supplementary material (Supplementary Table 1).

**Table 1:** Baseline characteristics.

Study	Study design	Shock type	AHDM device	Number of patients		Reported outcomes
				AHDM	Control	
Lu NF, 2015 [22]	RCT	Septic	PiCCO	53	52	1, 2, 3, 4, 6
Zhang Z, 2015 [14]	RCT	Septic	PiCCO	168	182	1, 2, 4
Richard JC, 2015 [23]	RCT	Septic	PiCCO	30	30	1, 2, 3, 4, 6
Lu X, 2022 [24]	Observational	Septic	PiCCO	200	200	1, 3, 4, 5
Sionis A, 2019 [25]	Observational	Cardiogenic	PAC	82	137	1, 2, 3, 5
Sotomi Y, 2014 [26]	Observational (PSM)	Cardiogenic	PAC	502	502	1, 2
Rossello X, 2016 [27]	Observational	Cardiogenic	PAC	83	46	1, 2
Kovács E, 2021 [28]	Observational	Cardiogenic	PiCCO	33	30	1, 2
Costa YC, 2024 [29]	Observational	Cardiogenic	PAC	37	69	1, 2
Costa YC, 2016 [30]	Observational	Cardiogenic	PAC	73	92	1
McKinley B, 2009 [31]	Observational	Mixed	PAC	79	103	1, 4, 6
Garan AR, 2020 [32]	Observational	Cardiogenic	PAC	598	260	1, 2
Arai R, 2024 [33]	Observational	Cardiogenic	PAC	1358	705	1, 2
Richard C, 2003 [34]	RCT	Mixed	PAC	335	341	1
Diaz-Arocutipa C, 2024 [21]	Observational (W)	Cardiogenic	PAC	53330	250640	1, 2, 5
Adler C, 2012 [35]	Observational	Cardiogenic	PiCCO	23	28	1, 2, 3, 5, 6
Sato R, 2024 [36]	Observational	Cardiogenic	PAC	1935	55800	1, 2, 3, 5
Réa AB, 2023 [37]	Observational	Cardiogenic	PAC	488	555	1, 2, 4
Kadosh B, 2023 [38]	Observational	Mixed	PAC	2719	10899	1
Ismayl M, 2023 [39]	Observational (PSM)	Cardiogenic	PAC	4700	4700	1, 2, 5
Kanwar M, 2023 [40]	Observational	Cardiogenic	PAC	834	221	1, 2, 5
Ni X, 2023 [41]	Non-randomised	Trauma- related	PiCCO	39	39	1
Luo Y, 2023 [42]	Observational	Septic	PiCCO	40	40	1, 2, 3, 4, 6
Watanabe A, 2024 [43]	Observational (PSM)	Cardiogenic	PAC	4495	4530	1, 2, 5
Hernandez G, 2019 [44]	Observational (PSM)	Cardiogenic	PAC	79682	835734	1, 2, 5
You J, 2024 [45]	Observational (PSM)	Cardiogenic	PiCCO	100	100	1, 2, 3, 5
Lian H, 2018 [46]	RCT	Septic	PiCCO	69	68	1, 3, 4
Wei W, 2020 [47]	Non-randomised	Septic	PiCCO	43	51	1, 2, 3, 4, 6
Ma S, 2017 [48]	RCT	Septic	PiCCO	20	20	1, 2, 3, 4, 6
Xu YH, 2011 [49]	Non-randomised	Septic	PiCCO	17	16	1, 6
Min Y, 2015 [50]	Observational	Septic	PiCCO	15	21	1, 3, 4
Lewejohann JC, 2015 [51]	Observational	Septic	PAC	10	10	1, 2
Rhodes A, 2002 [52]	RCT	Mixed	PAC	96	105	1, 2, 4, 5, 6
Ranka S, 2021 [53]	Observational (PSM)	Cardiogenic	PAC	25840	210316	1, 2, 5

PSM: propensity score-matched patients, W: weighted data.

1: mortality (in-hospital or 30-day mortality), 2: need for organ support (number of patients with inotropic-, vasopressor support, non-invasive and mechanical ventilation, renal replacement therapy and mechanical circulatory support [including IABP and VAD]) 3: duration of mechanical ventilation, 4: length of ICU stay, 5: length of hospital stay, 6: fluid amounts administered.

### Results of syntheses

**Fig. 2:**
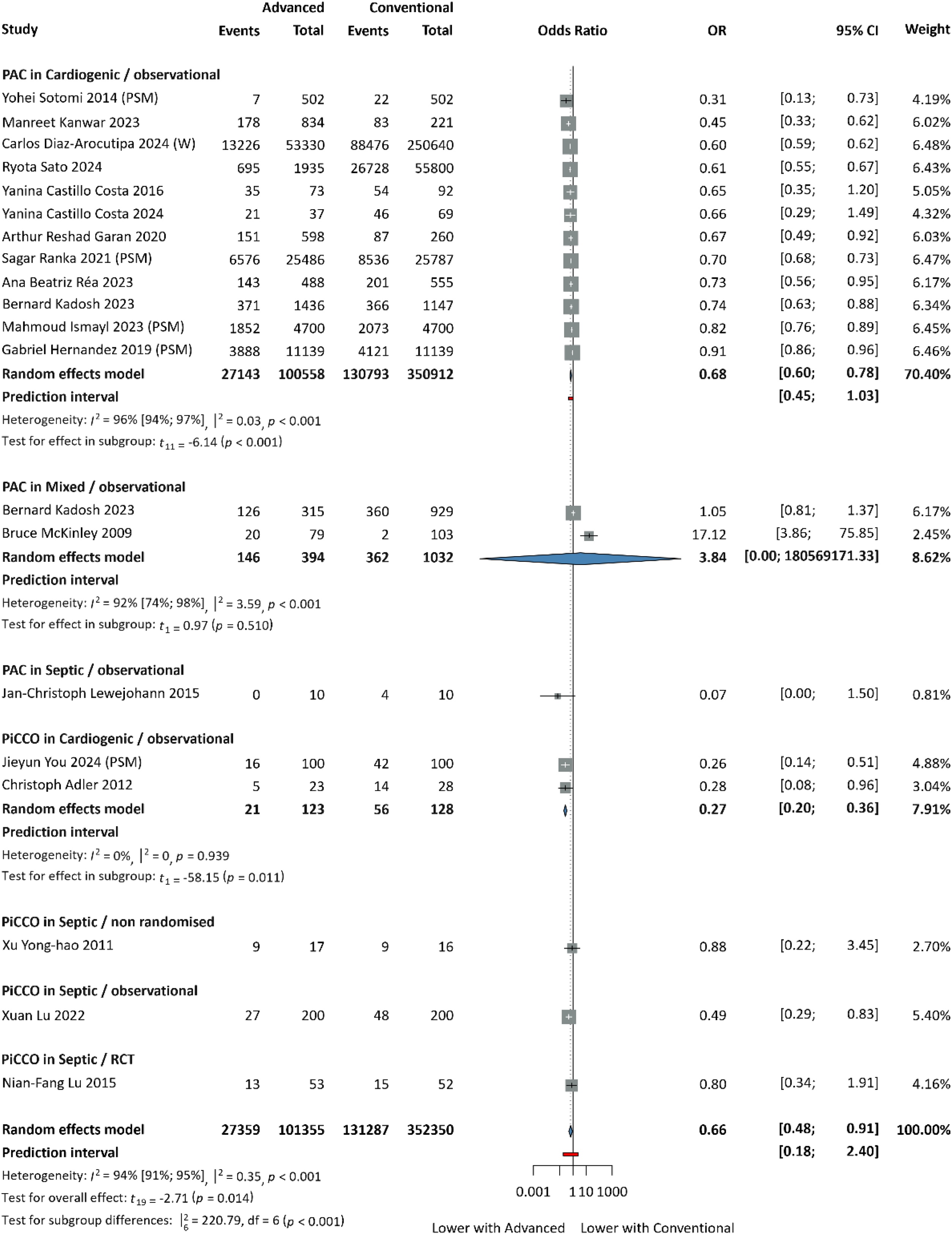
In-hospital mortality. PSM: propensity score matched patients; W: weighted data.

The primary outcome was mortality, assessed as in-hospital mortality (from admission to hospital discharge) and 30-day mortality (from admission to day 30). In terms of in-hospital mortality, our analysis included 19 studies, involving 453,705 patients. The odds of in-hospital mortality were significantly lower with AHDM compared with conventional methods (OR: 0.66; 95% CI: [0.48; 0.91], *p* < 0.001; Fig. 2). Subgroup analysis revealed a statistically significant reduction in in-hospital mortality in cardiogenic shock patients monitored with PAC (OR: 0.68, 95% CI: [0.60; 0.78], *p* < 0.001) and PiCCO (OR: 0.27, 95% CI: [0.20; 0.36], *p* = 0.011), compared with conventional methods or monitoring.

For 30-day mortality, our analysis included 16 studies, involving 12,936 patients. Although the point estimate favoured AHDM modestly, there was no difference in the odds of 30-day mortality between the two groups (OR: 0.89; 95% CI: [0.74; 1.07], *p* = 0.212).

**Fig. 3:**
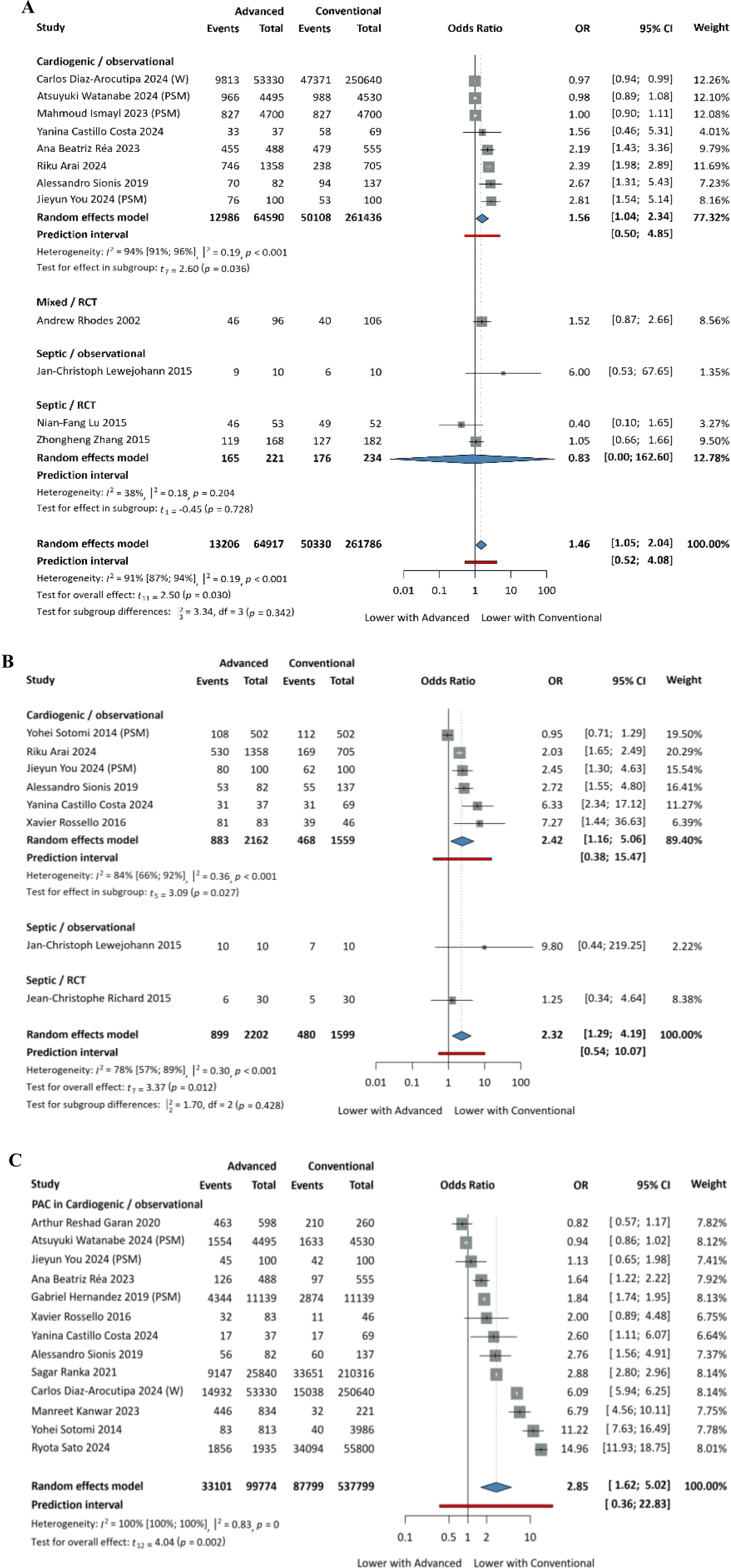
A: Need for vasopressor support; B: Need for inotropic support; C: Need for mechanical circulatory support. PSM: propensity score matched patients; W: weighted data.

**Fig. 4:**
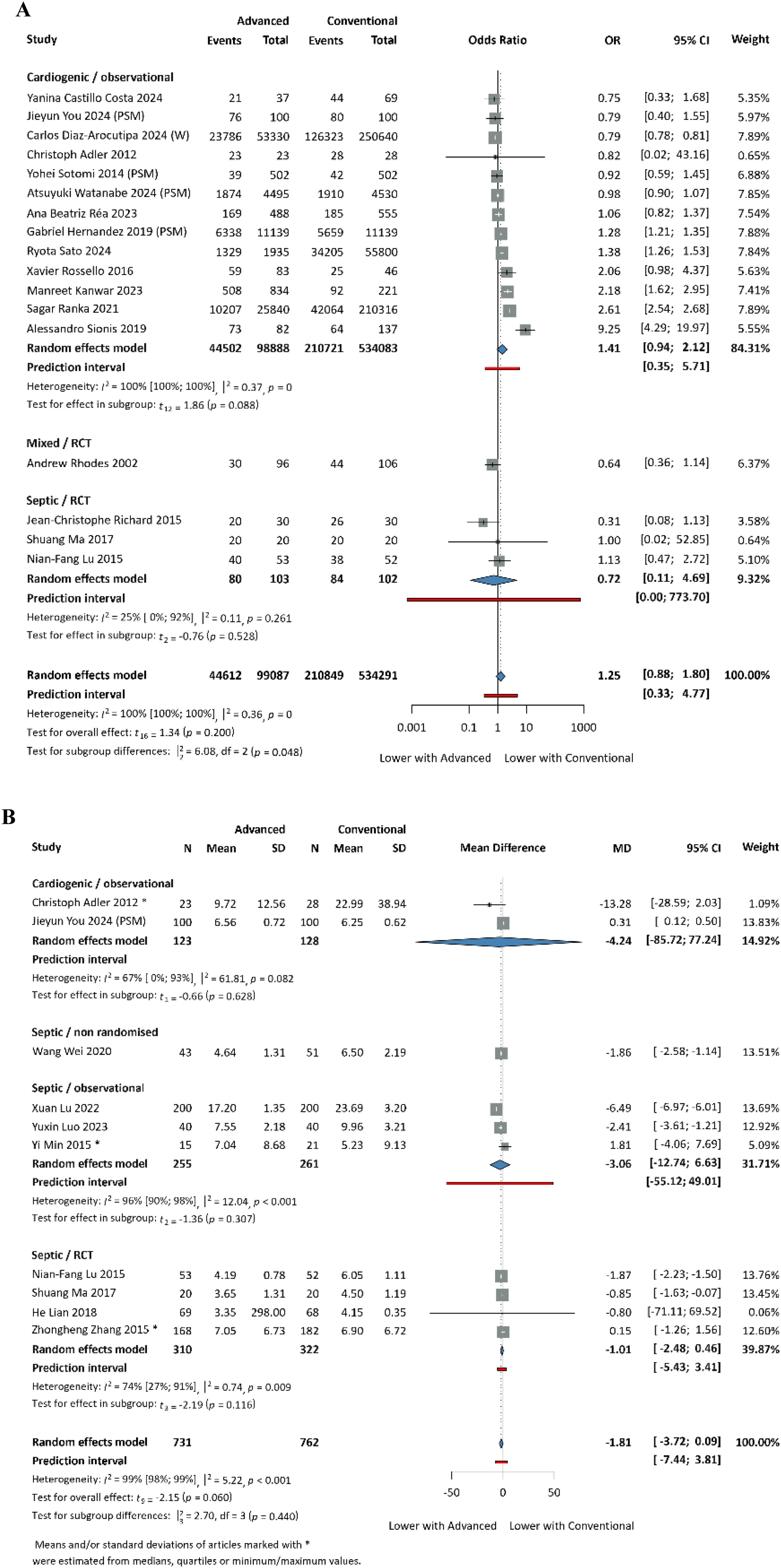
A: Need for mechanical ventilation; B: Length of mechanical ventilation (in days). PSM: propensity score matched patients; W: weighted data.

The need for various types of organ support - inotropic and vasopressor support, non-invasive and mechanical ventilation, renal replacement therapy and mechanical circulatory support (including IABP and VAD; evaluated only in cardiogenic shock population with PAC-guided AHDM) - was assessed in a cohort of 645,253 patients. Overall, patients managed with AHDM required more frequent use of nearly all forms of organ support, as shown in Table 2 and Fig. 3, Fig. 4.

**Table 2:** Results of the need for various types of organ support.

Organ support needed	Articles pooled	Number of patients	Pooled effect size
Inotropic support	8	3,801	OR: 2.32, CI: [1.29; 4.19]
Vasopressor support	12	326,703	OR: 1.46, CI: [1.05; 2.04]
Mechanical ventilation	17	633,378	OR: 1.25, CI: [0.88; 1.80]
Non-invasive ventilation	4	2,395	OR: 0.95, CI: [0.70; 1.28]
Renal replacement therapy	15	645,253	OR: 1.39, CI: [1.00; 1.93]
Mechanical circulatory support	13	637,573	OR: 2.85, CI: [1.62; 5.02]

Our analysis showed no statistically significant difference in the duration of mechanical ventilation, with a tendency towards a lower duration (MD: -1.81 days, 95% CI: [-3.72; 0.09]) in the AHDM group. We were unable to meta-analyse the duration of other organ-support therapies, due to the low number of eligible studies; therefore, we included these outcomes only in the systematic review.

The length of ICU stay, reported in 12 articles involving 2,301 patients, did not differ between the two groups (MD: -0.26 days, 95% CI: [-3.24; 2.72]. Differences in total hospital length of stay, analysed across 11 studies involving 640,639 patients, did not reach statistical significance (MD: 2.82 days, 95% CI: [-0.20; 5.84]).

The amount of fluids administered was analysed at six time points, as interval-specific volumes (0−6 h, 24−48 h and 48−72 h) and cumulative volumes (0−24 h, 0−48 h and 0−72 h). Presented in Table 3, none of these differences between the two groups reached statistical significance. The hemodynamic parameters used to guide fluid therapy are shown for each study in the corresponding forest plots.

**Table 3:** Fluid volumes administered at different time points.

Time points		Articles pooled	Number of patients	Pooled effect size
Interval- specific	0−6 h	7	594	MD: -73.92 ml, CI: [-655.70; 509.86]
	24−48 h	4	391	MD: -341.13 ml, CI: [-1378.85; 696.60]
	48−72 h	4	377	MD: -129.79 ml, CI: [-1068.32; 808.74]
Cumulative	0−24 h	6	631	MD: -290.25 ml, CI: [-1208.66; 628.17]
	0−48 h	2	134	MD: -1606.28 ml, CI: [-3441.90; 229.35]
	0−72 h	2	71	MD: -667.19 ml, CI: [-2551.54; 1217.16]

Data on organ dysfunction scores (SOFA, APACHE II) and complications related to the intervention were collected but could not be meta-analysed due to inconsistent reporting across studies. Reported adverse events were largely infrequent and limited to minor, reversible events; serious complications occurred only exceptionally, and no fatalities were attributed to the device.

Substantial heterogeneity was observed across studies, likely due to the inclusion of diverse shock types (cardiogenic, septic, trauma-related and mixed), different monitoring devices (PAC and PiCCO), and varying study designs. Subgroup analyses were therefore used to account for these differences.

### Risk of bias in studies, certainty in evidence

As detailed in Table 4, of the RCTs, three studies were judged to have a low risk of bias, two were rated as ‘some concerns’ and two as high risk, mainly due to deviations from the intended interventions (RoB 2, Domain 2). In contrast, all non-randomised studies were judged to have a high risk of bias, primarily due to confounding (ROBINS-I, Domain 2), as patient characteristics influenced the allocation of intervention. The certainty of evidence ranged from low to high across outcomes, with downgrading mainly due to risk of bias. A substantial heterogeneity was anticipated, the certainty of evidence was downgraded for inconsistency where appropriate.

**Table 4:** Results of risk of bias assessment.

Visual analysis and Egger's test were conducted for outcomes for which ten or more studies were meta-analysed. No significant publication bias was detected for in-hospital mortality or 30-day mortality (Egger's test *p* = 0.99 and *p* = 0.33 respectively).

**Fig. 5:**
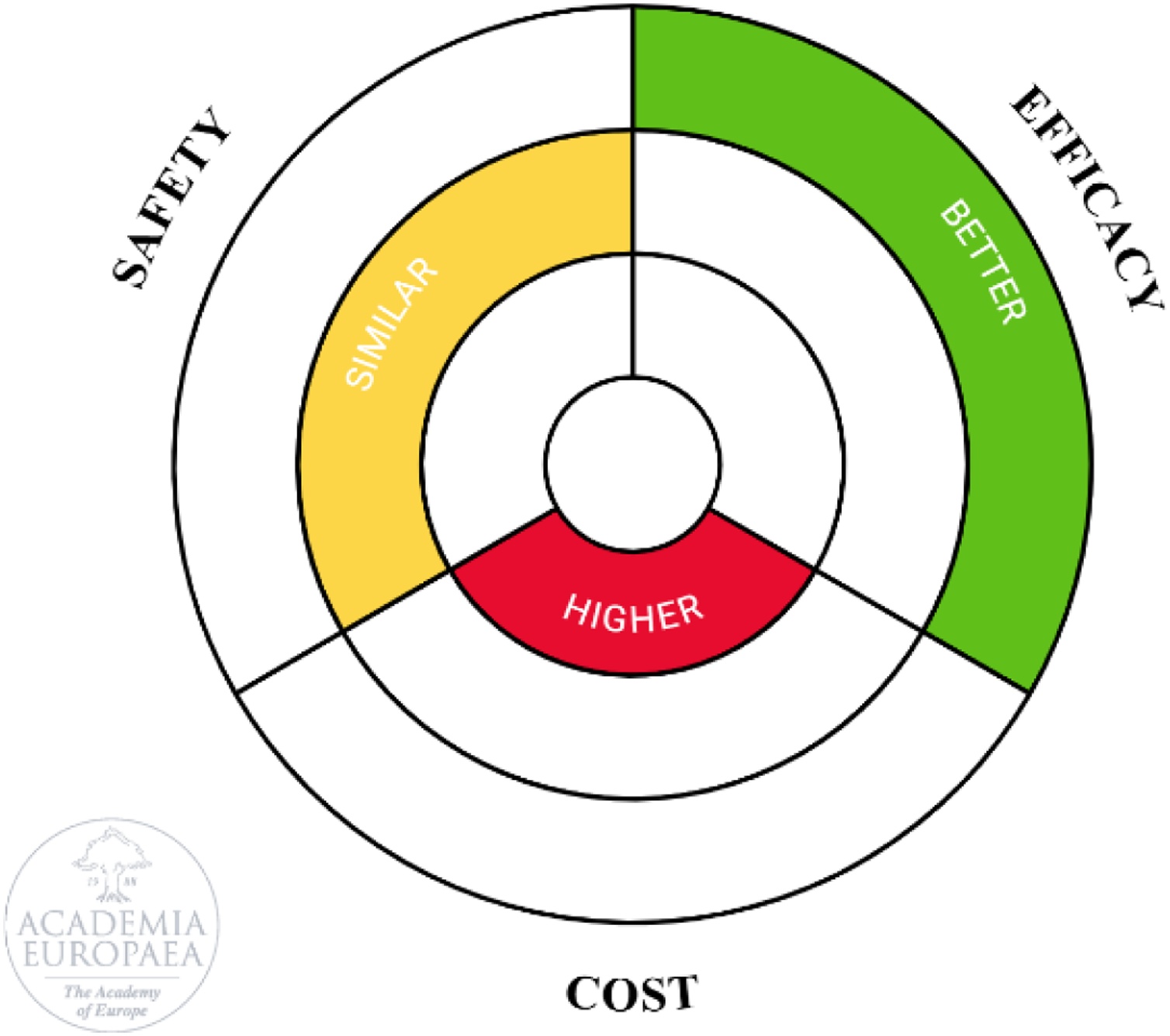
Ring diagram illustrating the efficacy, safety, and cost associated with the intervention.

A ring diagram was created based on three dimensions (Efficacy, Safety, Cost), within the cycle framework of the Academia Europaea [17]. As illustrated in Fig. 5, our results show that efficacy improved with AHDM, while safety was similar across groups, suggesting safer therapy guidance despite the intervention's invasiveness. Additionally, device-related costs were higher.

## Discussion

To the best of our knowledge, this is the most comprehensive systematic review and meta-analysis to date on the effects of AHDM in shock. Our results demonstrate that AHDM-guided shock management is associated with a significant reduction in in-hospital mortality, particularly in cardiogenic shock, with consistent findings across studies of PAC-based management.

### Interpretation of primary outcome

Our findings are consistent with most previous results, demonstrating a mortality benefit of PAC-guided management [[54], [55], [56], [57], [58]] and emphasising the importance of early initiation and complete hemodynamic profiling. Regarding PiCCO-guided decision-making, the available data, derived from a limited evidence base, suggest a hypothesis-generating benefit. However, a meta-analysis by Lee et al. found no significant mortality reduction with PAC [13]. In contrast, the modest mortality reduction we observed in septic shock was less pronounced than the 28-day mortality reported in the meta-analysis by Wang et al. [12]. In both cases, our inclusion of a larger number of more recent trials with greater patient populations provides more robust estimates that reflect contemporary treatment strategies. For mixed shock, no mortality reduction was found; whereas for trauma-related shock, which was supported only by one study, a potential survival gain could be observed. The overall findings suggest that AHDM can confer clinically meaningful benefits in shock management.

### Interpretation of secondary outcomes

Patients monitored with AHDM received inotropes, vasopressors, renal replacement therapy, and mechanical circulatory support more frequently; however, this higher escalation occurred in parallel with lower mortality. Although residual confounding by indication cannot be excluded, the observed pattern does not appear solely attributable to baseline severity differences, as such bias would likely disadvantage the AHDM group. In observational studies, higher rates of organ support may partly reflect clinicians’ tendency to escalate a greater investment in care for patients with a more favourable perceived prognosis, as well as a lower prevalence of treatment limitations in this group. The observed pattern suggests that AHDM might be associated with earlier recognition of circulatory failure and more timely, physiologically appropriate escalation of therapy, whereas conventional monitoring may delay detection and prolong conservative strategies. The requirements for inotropic and vasopressor support best reflect the severity of hemodynamic instability, suggesting that clinicians could target pharmacologic interventions more precisely. Data on mechanical circulatory support were available only in observational studies of cardiogenic shock, where its use was nearly threefold higher in the AHDM group compared with controls, in line with Bertina et al. [55]. The observed outcomes suggest that AHDM may facilitate qualitative changes in decision-making, consistent with precision-guided resuscitation.

In terms of the duration of mechanical ventilation, we observed a trend towards shorter ventilation in the AHDM group, which is consistent with the findings of Wang et al. [12]. The observed reduction of nearly two days, although not statistically significant, is clinically highly relevant and underscores the need for further studies in this field. Although this endpoint does not account for the competing risk of death, the lower mortality in the AHDM group supports its interpretation as a meaningful indicator of recovery rather than a survival surrogate.

Fluid administration patterns varied across studies. Although differences were small and not statistically significant, the trend suggests that AHDM may promote more careful resuscitation, thereby reducing the risk of fluid overload. This aligns with the findings of Scully et al., demonstrating a more favourable fluid balance with PiCCO [59]. Management was guided by multiple hemodynamic parameters, each demonstrating utility; however, no single parameter proved clearly superior. Heterogeneity in monitoring targets further limited comparability and may help explain the lack of a consistent statistical signal.

No significant differences were observed between the groups in terms of ICU and hospital stay. The slightly longer hospitalisation observed in the AHDM group may reflect improved survival and longer recovery in more severe conditions, rather than harm. However, Wang et al. reported shorter ICU stay with AHDM, in the PiCCO-guided group; hence, this topic is also worth investigating in the future [12].

Organ dysfunction scores (SOFA, APACHE II) and device-related complications could not be synthesised due to inconsistent reporting. Organ dysfunction scores were reported either only at admission or at varying time points, group comparisons a few days after therapy initiation were not feasible. Some observational studies reporting APACHE II scores [25,49,50] applied AHDM to patients with higher baseline severity, which may have resulted in biased comparisons. However, such confounding would likely underestimate the benefits of monitoring, as greater illness severity is intrinsically associated with poorer outcomes. In contrast, more observational studies [41,45,47,48,50] reported comparable baseline severity between groups, indicating no systematic imbalance. The overall pattern, therefore, aligns more coherently with a monitoring-driven shift toward earlier, physiologically informed, and more precisely targeted hemodynamic management rather than reflecting baseline risk differentials.

Reporting of complications associated with AHDM was also heterogeneous: some studies categorised complication types in detail, while others presented only aggregated outcomes, which limited the feasibility of generating clinically relevant pooled estimates.

### Strengths

This meta-analysis has several important strengths. To date, we have conducted the most comprehensive systematic search in multiple databases and have applied rigorous statistical methods. A large number of RCTs and observational studies were incorporated to capture real-world practice alongside trial-level data, and to cover diverse shock etiologies (including cardiogenic and trauma-related shock). Heterogeneity was addressed through predefined subgroup analyses by shock etiology, study design and device type. To date, we have also assessed the broadest range of clinical outcomes, providing a detailed evaluation supported by a moderate to high overall level of evidence. Furthermore, our findings are directly relevant to bedside decision-making, as they reflect contemporary clinical practice and are based on large-scale patient populations.

### Limitations

Despite these strengths, important limitations must be acknowledged. Evidence is dominated by observational studies, inherently prone to confounding and selection bias; furthermore, it is heavily weighted toward cardiogenic shock, limiting generalizability to other shock etiologies. Although subgroup analyses were performed, residual bias – including within-subgroup heterogeneity of shock phenotypes, potential treatment-escalation bias and variability in intervention thresholds across studies – cannot be excluded. No eligible studies evaluating LiDCO in shock populations were identified, limiting generalizability. Rare shock states such as neurogenic or prolonged anaphylactic shock were not represented, precluding conclusions for these populations. The inability to analyse organ dysfunction scores and device-related complications limited our insights into the mechanistic effects and safety of AHDM. Finally, although mortality was the most robust and clinically relevant endpoint, its dependence on multiple factors beyond hemodynamic monitoring renders it less sensitive to differences attributable solely to the intervention.

### Implications for clinical practice

Our findings have several implications for clinical practice. First, the data strongly support the use of AHDM in cardiogenic shock, particularly in patients requiring inotropic or mechanical circulatory support, providing crucial information for tailoring therapy and meaningfully improving survival. Second, although the benefit in septic shock is less certain, the absence of harm and the potential to reduce fluid overload suggest that AHDM may still have a role, especially in patients with complex hemodynamics or unclear fluid responsiveness. Third, the absence of significant differences in ICU stay and complications indicates that AHDM can be safely implemented without prolonging intensive care. Overall, despite higher device-related costs, AHDM appears to improve therapeutic efficacy without compromising safety [60], supporting its use as a tool for more precise and individualised management and encouraging clinicians to move beyond the “one-size-fits-all” approach. Instead, AHDM should be considered early in patients with cardiogenic shock, as well as in septic or mixed shock where hemodynamic uncertainty persists [56]. Importantly, device selection matters: our data suggest that both PAC and PiCCO are effective in cardiogenic shock, whereas evidence is lacking for LiDCO or minimally invasive technologies.

### Implications for future research

The translation of scientific evidence into routine clinical practice is crucial for enhancing patient care [17,60]. Further research is warranted to refine the role of AHDM in critical care. Well-designed RCTs are needed to confirm the survival benefit observed in cardiogenic shock and to clarify the utility in sepsis and trauma. To enhance comparability and translational impact, future studies should adopt standardised frameworks tailored to report organ dysfunction trajectories, device-related procedural complications and long-term outcomes. Future studies should establish the outcome relevance of specific and combined hemodynamic targets; and identify shock phenotypes that derive the greatest benefit, integrating hemodynamic data with biomarkers and advanced imaging. Currently, less invasive methods are associated with higher measurement error [10], highlighting the need for more precise technologies to enable safer application of AHDM in clinical practice.

## Conclusions

Clinical decision-making and hemodynamic management guided by advanced monitoring were associated with reduced in-hospital mortality, with the greatest benefit occurring among patients with cardiogenic shock. In patients monitored with AHDM, more frequent escalation of organ support was observed. Overall, our findings suggest that AHDM may facilitate qualitative differences in clinical decision-making, consistent with a more individualized, physiology-informed approach to resuscitation.

## CRediT author contribution statement

LN: conceptualisation, investigation, writing - original draft; PRT: investigation, writing – review and editing; CT: methodology, supervision, writing – review and editing; DL: methodology, supervision, writing – review and editing; LL: investigation, writing – review and editing; BD: investigation, writing – review and editing; ZS: formal analysis, statistics, writing – review and editing; PH: project administration, writing – review and editing; ZM: conceptualisation, supervision, writing – review and editing; LZ: conceptualisation, supervision, writing – review and editing

All authors confirm that they contributed substantially to the work and accept full responsibility for its content, including involvement in the study’s conception and design of the study, data analysis and manuscript writing or revision.

## Ethical approval

Ethical approval was not required for this systematic review and meta-analysis, as it was based solely on previously published data from peer-reviewed studies. No patients were directly involved in the design, conduct, or interpretation of the research. All datasets analysed are publicly available in the original full-text articles included in the review.

## Funding

This study was conducted at the Centre for Translational Medicine, Semmelweis University, with support from the Hungarian National Research, Development and Innovation Office [K138816].

## Declaration of competing interest

Zsolt Molnár receives honoraria for lectures and consulting from CytoSorbents Europe, ThermoFisher, Baxter, PULSION Medical (Getinge Group), Nutricia and Biotest.
