# Supplementary material for: Invasive hemodynamic monitoring-guided resuscitation improves survival in shock: A systematic review and meta-analysis
Source: Ann Intensive Care. 2026 Apr 29;16:100071. doi: 10.1016/j.aicoj.2026.100071 (PMC13141741; doi:10.1016/j.aicoj.2026.100071)
Supplement: Supplementary file 1 [file mmc1.docx]

Electronic Supplementary Material

[Search strategy 3](#_Toc226967891)

[Baseline Characteristics – Extended Table 5](#_Toc226967892)

[Data extraction table forms 8](#_Toc226967893)

[Forest plots not included in the main text 9](#_Toc226967894)

[Publication bias figures – Funnel plots 16](#_Toc226967895)

[Risk of bias assessment for each outcome 18](#_Toc226967896)

[Detailed results of GRADE assessment 20](#_Toc226967897)

[PRISMA reporting guidelines 24](#_Toc226967898)

# Search strategy

Prepared by: Liliána Nagy

Date of search: 09. 11. 2024.

Title: Investigating the efficacy and safety of advanced versus conventional hemodynamic monitoring in patients with shock

Clinical question: Is therapy guided by cardiac output (CO) measurement a safe and more efficient alternative to conventional one in patients in shock?

Framework: PICO

**P** patients in shock
**I** CO measurement-guided therapy
**C** standard therapy
**O_1_** mortality
**O_2_** organ support-free days, organ dysfunction scores, length of stay, safety

**Pubmed**

(("cardiogenic" OR "neurogenic" OR "traumatic" OR "hemorrhagic" OR "haemorrhagic" OR "hypovolemic" OR "distributive" OR "septic" OR "infectious" OR "anaphylactic" OR "obstructive") AND ("shock" OR "instability" OR "unstable”))

AND

((("intraarterial" OR "intravascular" OR "intravenous") AND (manag* OR monitor* OR measur*)) OR "PiCCO" OR (("pulse" AND "indicated" AND "continuous" AND "cardiac" AND "output") OR "LiDCO" OR ("Lithium" AND dilut* AND "cardiac" AND "output") OR "thermodilution" OR ("pulmonary" AND "artery" AND "catheter") OR "transpulmonary" OR ("Swan" AND "Ganz") OR "Swan-Ganz"))

Domain 1 refers to: population

Domain 2 refers to: intervention

Number of results: 3970

Notes: I used the truncation **manag*** and **monitor*** and **measur*** to find all varieties of the words, like measurement and measuring.

Database settings: no special settings

**Embase**

((cardiogenic OR neurogenic OR traumatic OR hemorrhagic OR haemorrhagic OR hypovolemic OR distributive OR septic OR infectious OR anaphylactic OR obstructive) AND (shock OR instability OR unstable))

AND

(((intraarterial OR intravascular OR intravenous) AND (manag* OR monitor* OR measur*)) OR PiCCO OR ((pulse AND indicated AND continuous AND cardiac AND output) OR LiDCO OR (Lithium AND dilut* AND cardiac AND output) OR thermodilution OR (pulmonary AND artery AND catheter) OR transpulmonary OR (Swan AND Ganz) OR Swan-Ganz)):ab,kw,ti

Domain 1 refers to: population

Domain 2 refers to: intervention

Number of results: 7061

Notes: I used the truncation **manag*** and **monitor*** and **measur*** to find all varieties of the words, like measurement and measuring. We searched for abstracts, keywords, titles only.

Database settings: we turned off all automatic mapping options

**CENTRAL (Cochrane Library)**

((cardiogenic OR neurogenic OR traumatic OR hemorrhagic OR haemorrhagic OR hypovolemic OR distributive OR septic OR infectious OR anaphylactic OR obstructive) AND (shock OR instability OR unstable))

AND

(((intraarterial OR intravascular OR intravenous) AND (manag* OR monitor* OR measur*)) OR PiCCO OR ((pulse AND indicated AND continuous AND cardiac AND output) OR LiDCO OR (Lithium AND dilut* AND cardiac AND output) OR thermodilution OR (pulmonary AND artery AND catheter) OR transpulmonary OR (Swan AND Ganz) OR Swan-Ganz))

Domain 1 refers to: population

Domain 2 refers to: intervention

Number of results: 1099

Notes: I used the truncation **manag*** and **monitor*** and **measur*** to find all varieties of the words, like measurement and measuring.

Database settings: we searched “All text” and filtered “Trials”

# Baseline Characteristics – Extended Table

| Study | Study design | Country | Centre / Database | Shock type | AHDM Device | Number of patients | | Gender  (Female / Male) | | Age | | Duration of the study | Reported outcomes |
| --- | --- | --- | --- | --- | --- | --- | --- | --- | --- | --- | --- | --- | --- |
|  |  |  |  |  |  | AHDM | Control | AHDM | Control | AHDM | Control |  |  |
| Lu NF,  2015 | RCT | China | Single-centre | Septic | PiCCO | 53 | 52 | 17 / 36 | 19 / 33 | 61 ± 15 | 62 ± 14 | 2008 - 2010 | 1, 2, 3, 4, 6 |
| Zhang Z,  2015 | RCT | China | Single-centre | Septic | PiCCO | 168 | 182 | 47 / 121 | 45 / 137 | 62 ± 16 | 65 ± 15 | 2012 - 2014 | 1, 2, 4 |
| Richard JC, 2015 | RCT | France | Single-centre | Septic | PiCCO | 30 | 30 | 9 / 21 | 8 / 22 | 65  [58-80] | 64  [54-76] | 2007 - 2013 | 1, 2, 3, 4, 6 |
| Lu X,  2022 | observational | China | Single-centre | Septic | PiCCO | 200 | 200 | 67 / 133 | 78 / 122 | 60 ± 5 | 60 ± 5 | 2018 - 2021 | 1, 3, 4, 5 |
| Sionis A,  2019 | observational | International | Multicentre | Cardiogenic | PAC | 82 | 137 | 18 / 64 | 69 / 68 | 65 ± 12 | 68 ± 11 | 2010 - 2012 | 1, 2, 3, 5 |
| Sotomi Y, 2014 | observational (PSM) | Japan | ATTEND Registry | Cardiogenic | PAC | 502 | 502 | 184 / 318 | 187 / 315 | 67 ± 14 | 68 ± 15 | 2007 - 2011 | 1, 2 |
| Rossello X, 2016 | observational | Spain | Single-centre | Cardiogenic | PAC | 83 | 46 | 29 / 54 | 16 / 30 | 66 ± 16 | 71 ± 13 | 2005 - 2009 | 1, 2 |
| Kovács E, 2021 | observational | Hungary | Single-centre | Cardiogenic | PiCCO | 33 | 30 | 6 / 27 | 13 / 17 | 65  [56-69] | 64  [56-71] | 2008 - 2015 | 1, 2 |
| Costa YC, 2024 | observational | Argentina | Multicentre | Cardiogenic | PAC | 37 | 69 | 8 / 29 | 23 / 46 | 62  [56-73] | 65  [58-74] | 2021 - 2022 | 1, 2 |
| Costa YC, 2016 | observational | Argentina | Multicentre | Cardiogenic | PAC | 73 | 92 | 58 / 107 | | 66 [58-76.5] | | 2013 - 2015 | 1 |
| McKinley B, 2009 | observational | United States | Single-centre | Mixed | PAC | 79 | 103 | 22 / 57 | 24 / 79 | 37 ± 2 | 39 ± 1 | 2004 - 2006 | 1, 4, 6 |
| Garan AR, 2020 | observational | United States | Multicentre | Cardiogenic | PAC | 598 | 260 | 182 / 416 | 64 / 196 | 59 ± 16 | 63 ± 16 | 2016 - 2019 | 1, 2 |
| Arai R,  2024 | observational | Japan | Multicentre | Cardiogenic | PAC | 1358 | 705 | 310 / 1048 | 162 / 543 | NA | NA | 2020 - 2021 | 1, 2 |
| Richard C, 2003 | RCT | France | Multicentre | Mixed | PAC | 335 | 341 | 111 / 224 | 113 / 228 | 62 ± 16 | 63 ± 15 | 1999 - 2001 | 1 |
| Study | Study design | Country | Centre / Database | Shock type | AHDM Device | Number of patients | | Gender  (Female / Male) | | Age | | Duration of the study | Reported outcomes |
|  |  |  |  |  |  | AHDM | Control | AHDM | Control | AHDM | Control |  |  |
| Diaz-Arocutipa C, 2024 | observational (W) | Spain | National Inpatient Sample database | Cardiogenic | PAC | 53330 | 250640 | 17915 / 35415 | 100615 / 150025 | 63  [53-72] | 69  [58-78] | 2017-2019 | 1, 2, 5 |
| Adler C,  2012 | observational | Germany | Single-centre | Cardiogenic | PiCCO | 23 | 28 | 6 / 17 | 2 / 26 | 60  (34-89) | 62  (28-80) | 2007 - 2010 | 1, 2, 3, 5, 6 |
| Sato R,  2024 | observational | United States | National Inpatient Sample database | Cardiogenic | PAC | 1935 | 55800 | 840 / 1095 | 24162 / 31638 | 62 (SEM: 0.75) | 69 (SEM: 0.15) | 2017 - 2019 | 1, 2, 3, 5 |
| Réa AB,  2023 | observational | Canada | Single-centre | Cardiogenic | PAC | 488 | 555 | 131 / 357 | 184 / 371 | 60 ± 14 | 62 ± 17 | 2014 - 2020 | 1, 2, 4 |
| Kadosh B, 2023 | observational | International | Critical Care Cardiology Trials Network | Mixed | PAC | 2719 | 10899 | 876 / 1843 | 4156 / 6743 | 67  [57-77] | 64  [54-72] | 2017 - 2021 | 1 |
| Ismayl M, 2023 | observational (PSM) | United States | National Inpatient Sample database | Cardiogenic | PAC | 4700 | 4700 | 1278 / 3422 | 1241 / 3459 | 65  [57-73] | 65  [57-73] | 2016 - 2020 | 1, 2, 5 |
| Kanwar M, 2023 | observational | United States | Multicentre, Cardiogenic Shock Working Group registry | Cardiogenic | PAC | 834 | 221 | 225 / 609 | 94 / 127 | 61 ± 14 | 67 ± 15 | 2019 - 2021 | 1, 2, 5 |
| Ni X,  2023 | non-randomised | China | Single-centre | Trauma- related | PiCCO | 39 | 39 | 12 / 27 | 14 / 25 | 49 ± 6 | 48 ± 5 | 2018 - 2020 | 1 |
| Luo Y,  2023 | observational | China | Single-centre | Septic | PiCCO | 40 | 40 | 14 / 26 | 12 / 28 | 57 ± 4 | 57 ± 4 | 2018 - 2020 | 1, 2, 3, 4, 6 |
| Watanabe A, 2024 | observational (PSM) | United States | Multicentre | Cardiogenic | PAC | 4495 | 4530 | 1484 / 3011 | 1498 / 3032 | 73  [69-78] | 73  [69-78] | 2016 - 2020 | 1, 2, 5 |
| Study | Study design | Country | Centre / Database | Shock type | AHDM Device | Number of patients | | Gender  (Female / Male) | | Age | | Duration of the study | Reported outcomes |
|  |  |  |  |  |  | AHDM | Control | AHDM | Control | AHDM | Control |  |  |
| Hernandez G, 2019 | observational (PSM) | International | National Inpatient Sample database | Cardiogenic | PAC | 79682 | 835734 | 28845 / 50837 | 340144 / 495590 | 64 ± 15 | 68 ± 15 | 2004 - 2014 | 1, 2, 5 |
| You J,  2024 | observational (PSM) | China | Single-centre | Cardiogenic | PiCCO | 100 | 100 | 26 / 74 | 30 / 70 | 71 ± 12 | 72 ± 14 | 2022 - 2023 | 1, 2, 3, 5 |
| Lian H,  2018 | RCT | China | Single-centre | Septic | PiCCO | 69 | 68 | 30 / 39 | 31 / 37 | 54 ± 10 | 52 ± 6 | 2015 - 2017 | 1, 3, 4 |
| Wei W,  2020 | non-randomised | China | Multicentre | Septic | PiCCO | 43 | 51 | 20 / 23 | 18 / 33 | 66 ± 11 | 65 ± 12 | 2017 - 2020 | 1, 2, 3, 4, 6 |
| Ma S,  2017 | RCT | China | Single-centre | Septic | PiCCO | 20 | 20 | 5 / 15 | 6 / 14 | 78 ± 7 | 77 ± 7 | 2013 - 2015 | 1, 2, 3, 4, 6 |
| Xu YH,  2011 | non-randomised | China | Single-centre | Septic | PiCCO | 17 | 16 | 7 / 10 | 1 / 15 | 64 ± 11 | 71 ± 10 | 2008 - 2009 | 1, 6 |
| Min Y,  2015 | observational | China | Single-centre | Septic | PiCCO | 15 | 21 | 6 / 9 | 13 / 8 | 68 ± 18 | 70 ± 17 | 2011 - 2013 | 1, 3, 4 |
| Lewejohann JC, 2015 | observational | Germany | Single-centre | Septic | PAC | 10 | 10 | 3 / 7 | 2 / 8 | NA | NA | 3 years and 7 months | 1, 2 |
| Rhodes A, 2002 | RCT | England | Single-centre | Mixed | PAC | 96 | 105 | NA | NA | 68  [52-74] | 64  [49-73] | 1997 - 1999 | 1, 2, 4, 5, 6 |
| Ranka S,  2021 | observational (PSM) | United States | Nationwide Readmissions Database | Cardiogenic | PAC | 25840 | 210316 | 8192 / 17648 | 81603 / 128713 | 62 ± 15 | 67 ± 14 | 2016 - 2017 | 1, 2, 5 |

Supplementary Table 1. Baseline characteristics
Data are presented as reported in the original studies (mean ± SD, median [IQR], mean (range), or mean (SEM)). Mean (SEM) values are indicated in the table.
PSM: propensity score–matched patients; W: weighted data; SEM: standard error of the mean; NA: not available.
1: mortality (in-hospital or 30-day); 2: need for organ support (number of patients receiving inotropic or vasopressor support, non-invasive or invasive mechanical ventilation, renal replacement therapy, or mechanical circulatory support [including IABP and VAD]); 3: duration of mechanical ventilation; 4: length of ICU stay; 5: length of hospital stay; 6: fluid volume administered.

# Data extraction table forms

The data extraction table forms (Supplementary Tables 1-3) were completed for each included study across all outcomes. Each table comprises a study characteristics section, followed by the relevant outcome table, depending on whether the outcome is dichotomous or continuous.

Study characteristics

| Publication data | | | | | Population | Intervention | | Control / Comparator | |
| --- | --- | --- | --- | --- | --- | --- | --- | --- | --- |
| First Author | Year of publication | DOI | Study type | Study period | Population details | Monitoring device | Details | Monitoring device | Details |
|  |  |  |  |  |  |  |  |  |  |

Outcome: Dichotomous outcome

| Outcome data | | | | | Duration of follow-up | | | | | | |
| --- | --- | --- | --- | --- | --- | --- | --- | --- | --- | --- | --- |
| Details | Event in AHDM group | Total patients in AHDM group | Event in control group | Total patients in control group | Mean | Median | Standard deviation | IQR min | IQR max | Range min | Range max |
|  |  |  |  |  |  |  |  |  |  |  |  |

Outcome: Continuous outcome

|  | AHDM group | | | | | | | | | | | Control group | | | | | | | | | | |
| --- | --- | --- | --- | --- | --- | --- | --- | --- | --- | --- | --- | --- | --- | --- | --- | --- | --- | --- | --- | --- | --- | --- |
| Details | N0 of patients | Mean | Median | Standard deviation | Standard error | CI min | CI max | IQR min | IQR max | Range min | Range max | N0 of patients | Mean | Median | Standard deviation | Standard error | CI min | CI max | IQR min | IQR max | Range min | Range max |
|  |  |  |  |  |  |  |  |  |  |  |  |  |  |  |  |  |  |  |  |  |  |  |

Supplementary Tables 2-4. Data extraction tables

# Forest plots not included in the main text
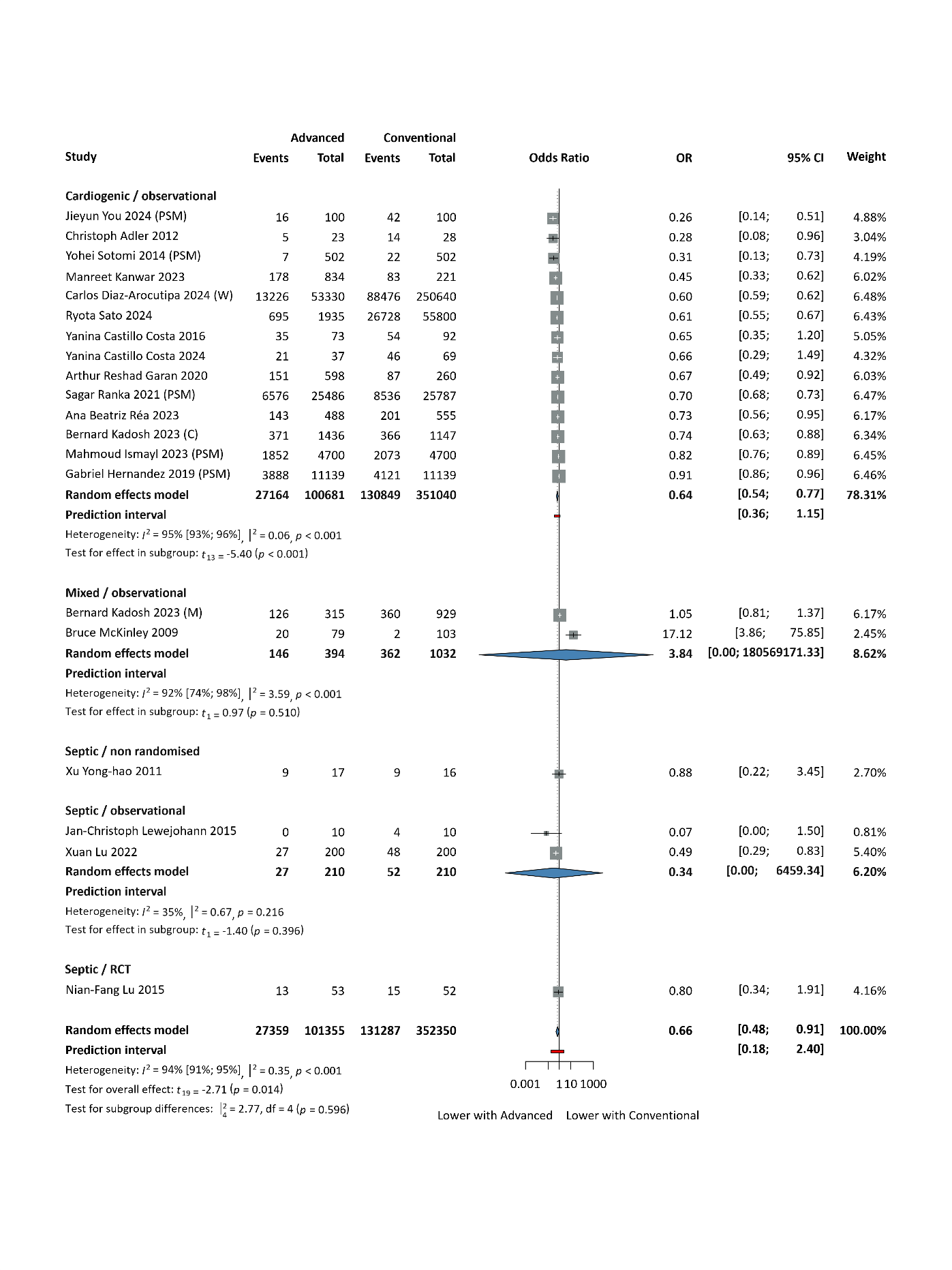


Supplementary Figure 1. In-hospital mortality.
 Subgroups based on shock aetiology and study design
 PSM: propensity score matched patients, W: weighted data


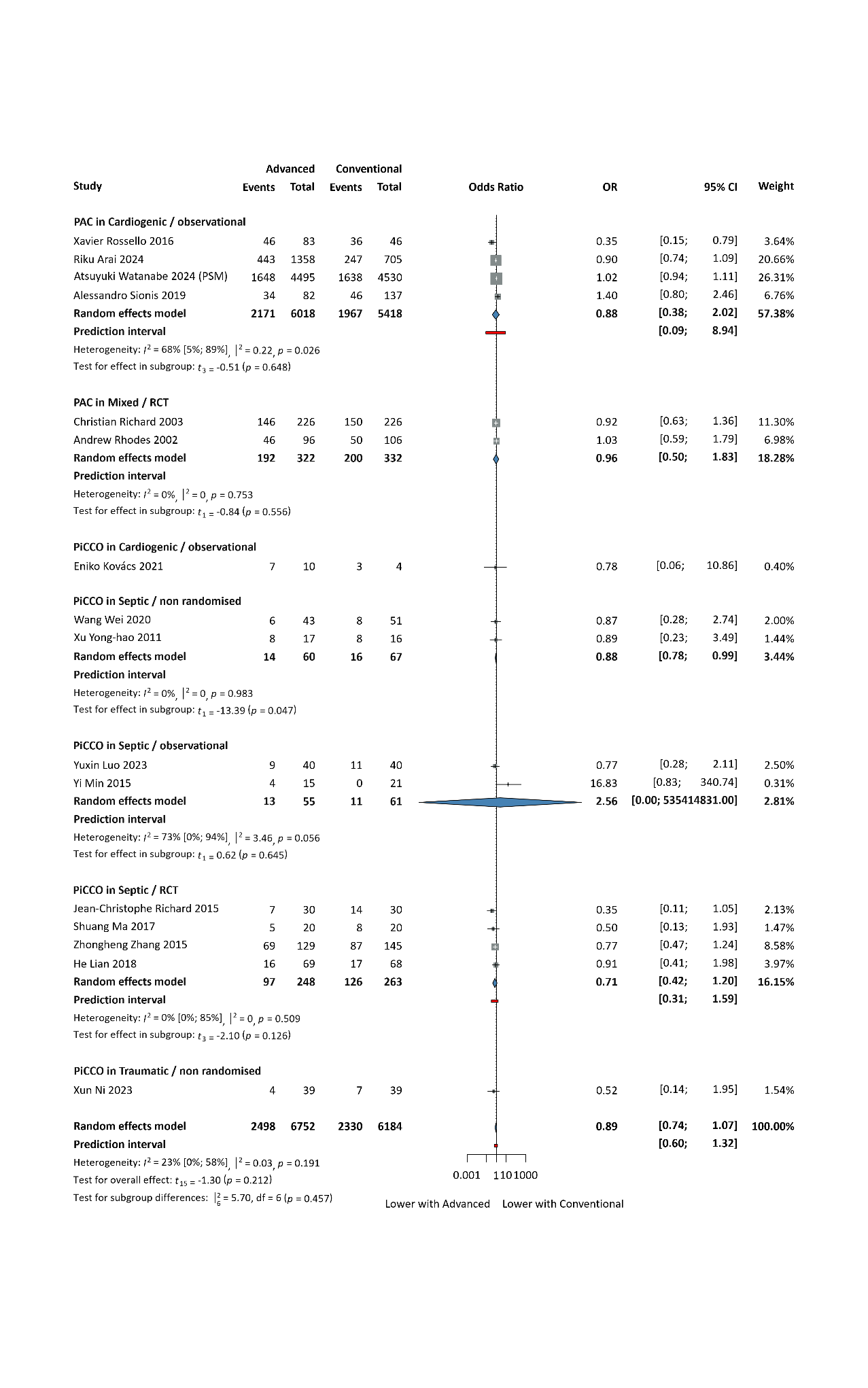


Supplementary Figure 2. 30-day Mortality
Subgroups based on shock aetiology, study design and monitoring device
PSM: propensity score matched patients


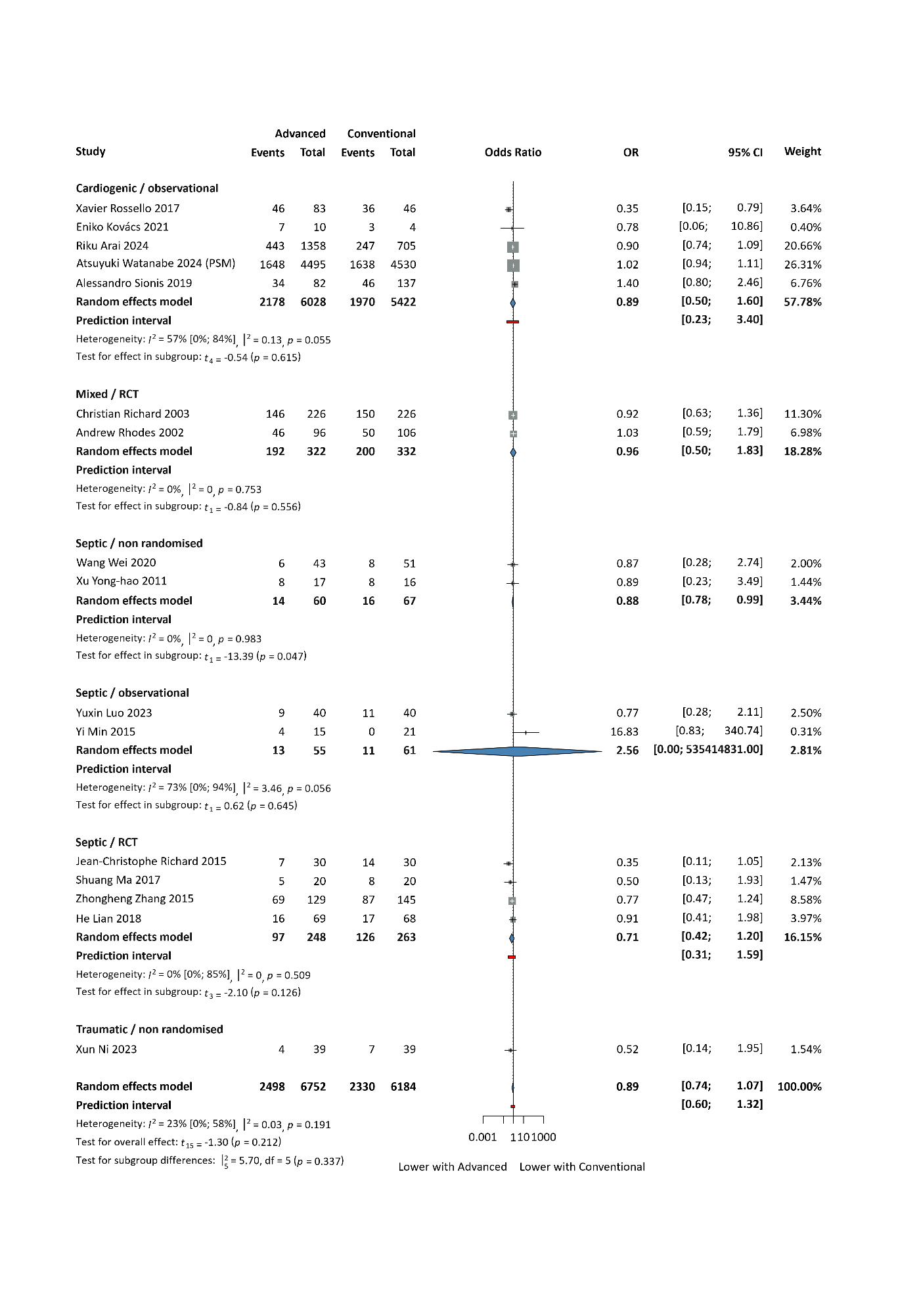


Supplementary Figure 3. 30-day mortality
Subgroups based on shock aetiology and study design
PSM: propensity score matched patients, W: weighted data

| 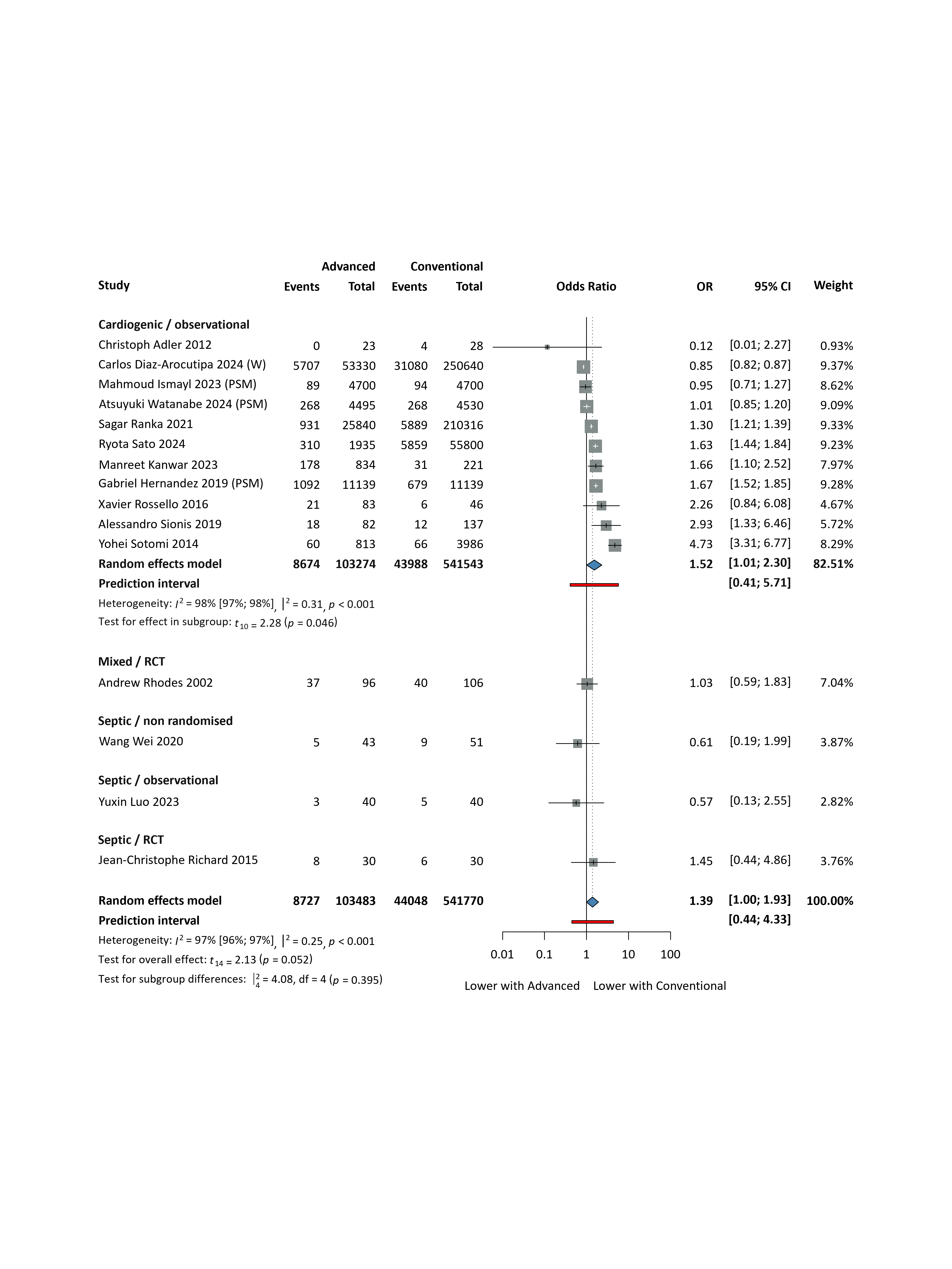  Supplementary Figure 4. Need for renal replacement therapy |
| --- |
| 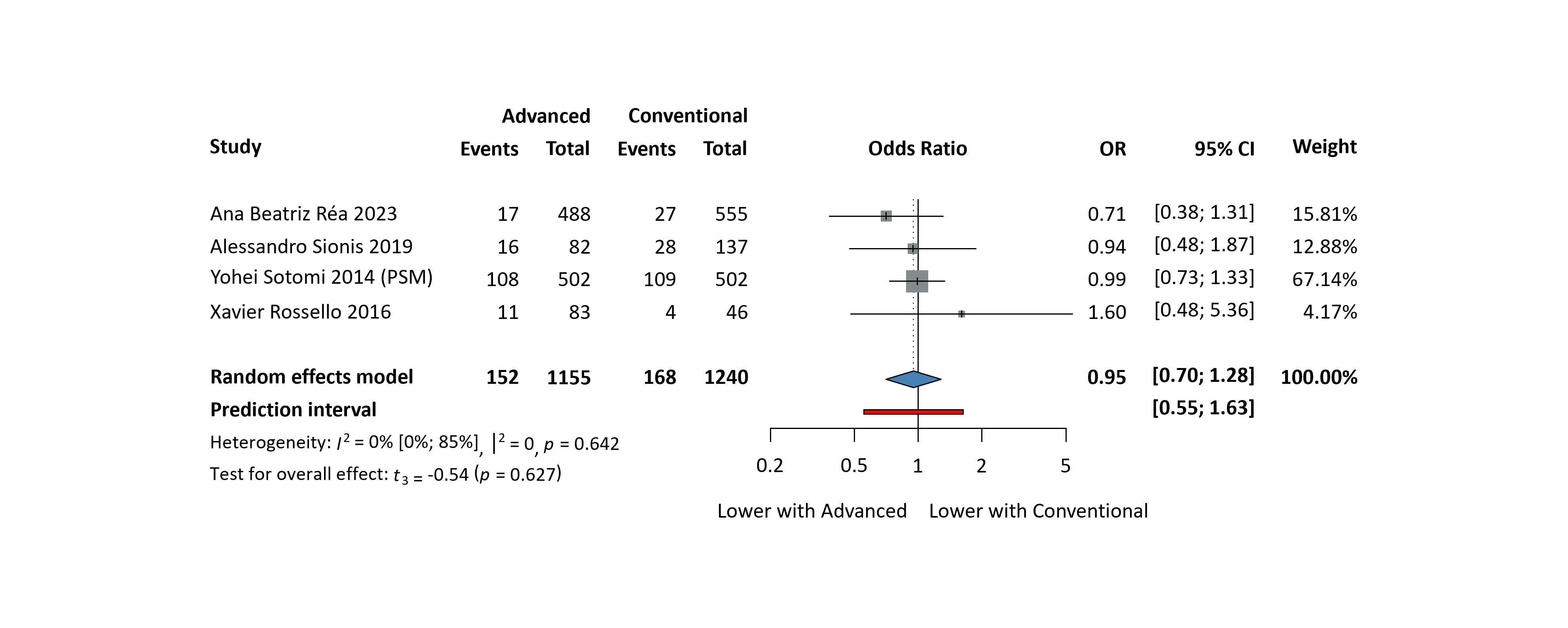  Supplementary Figure 5. Need for non-invasive ventilation |
|  |
| 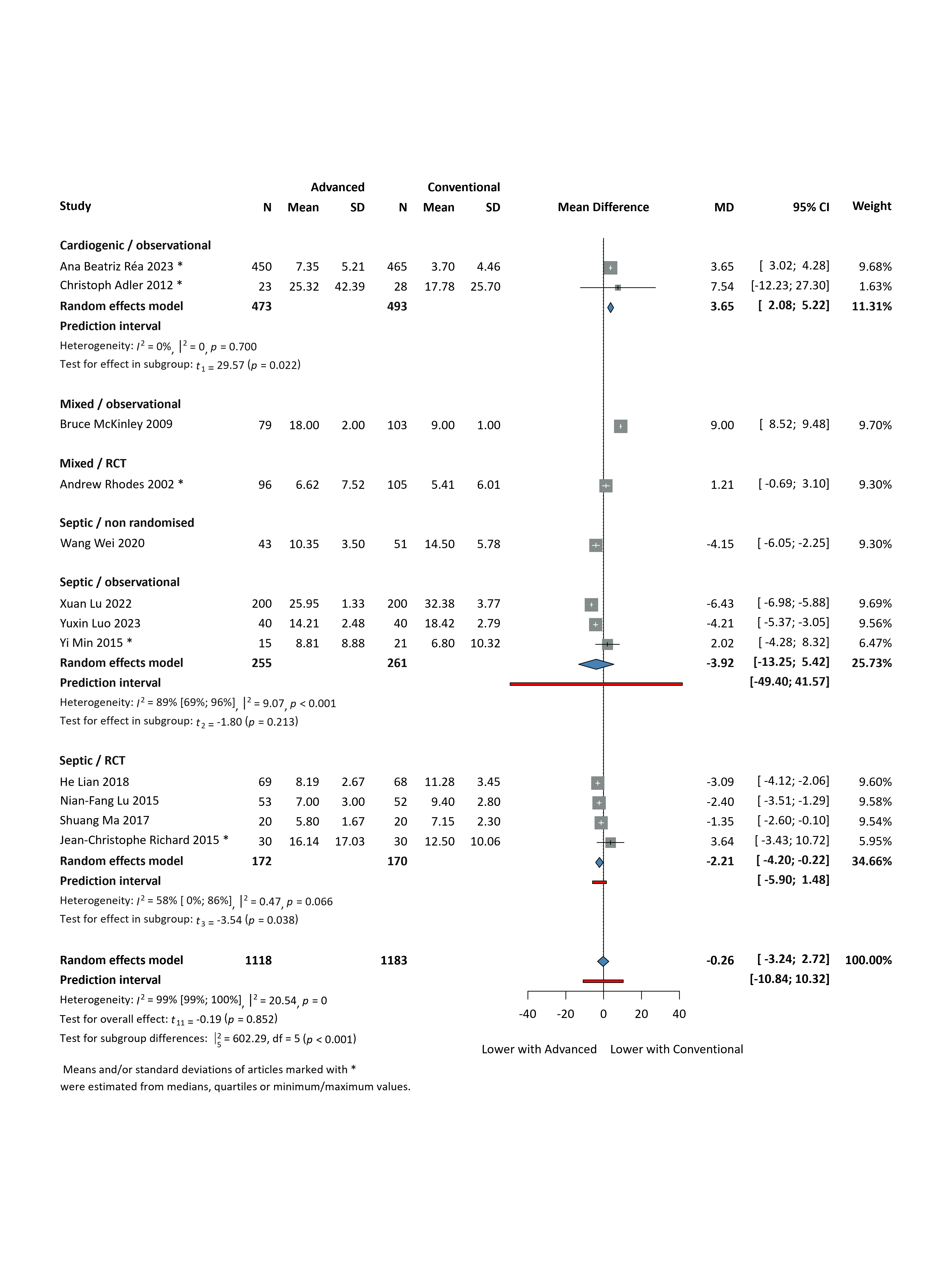  Supplementary Figure 6. Length of ICU-stay |
|  |
| 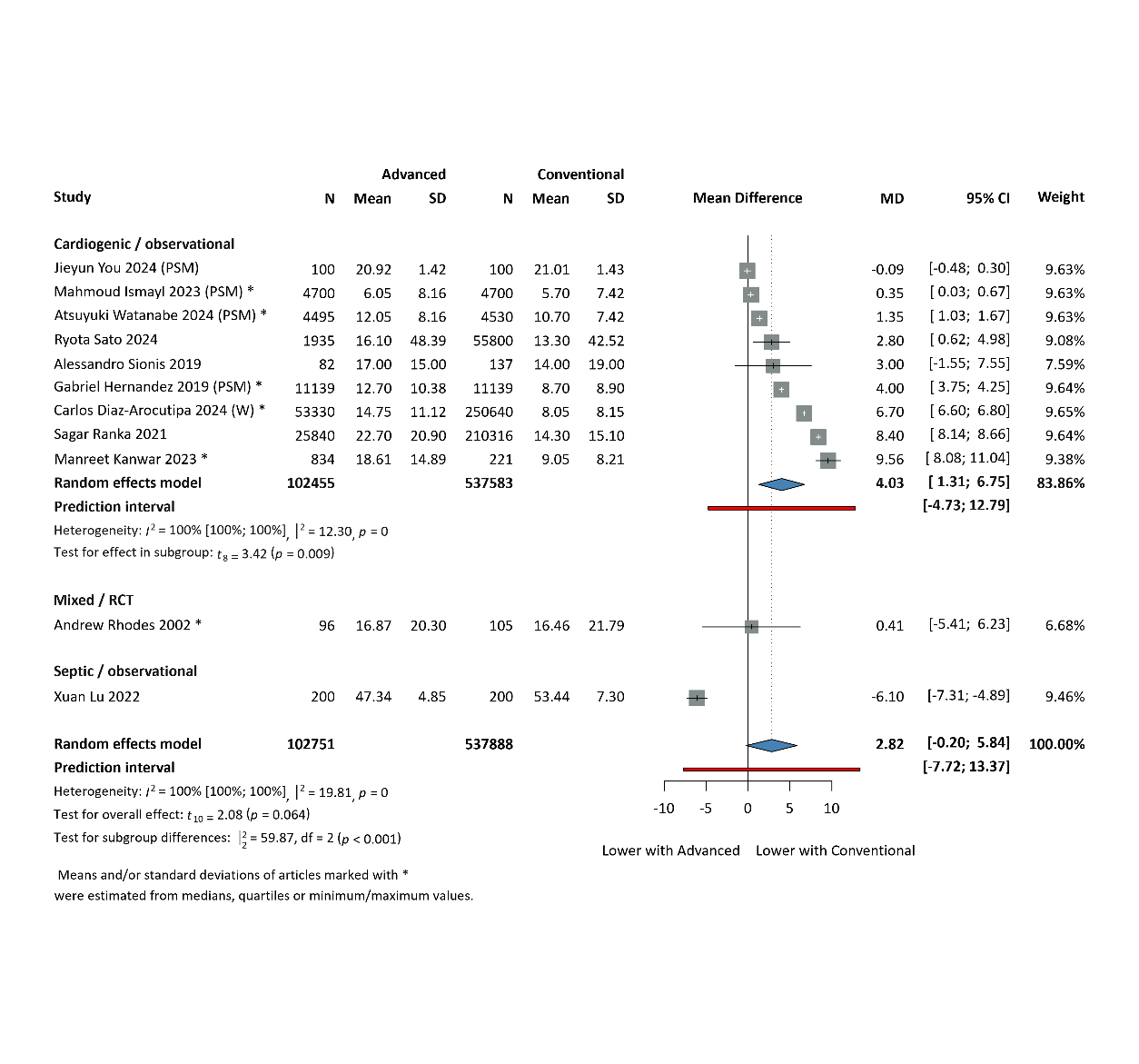  Supplementary Figure 7. Length of hospital stay |
| 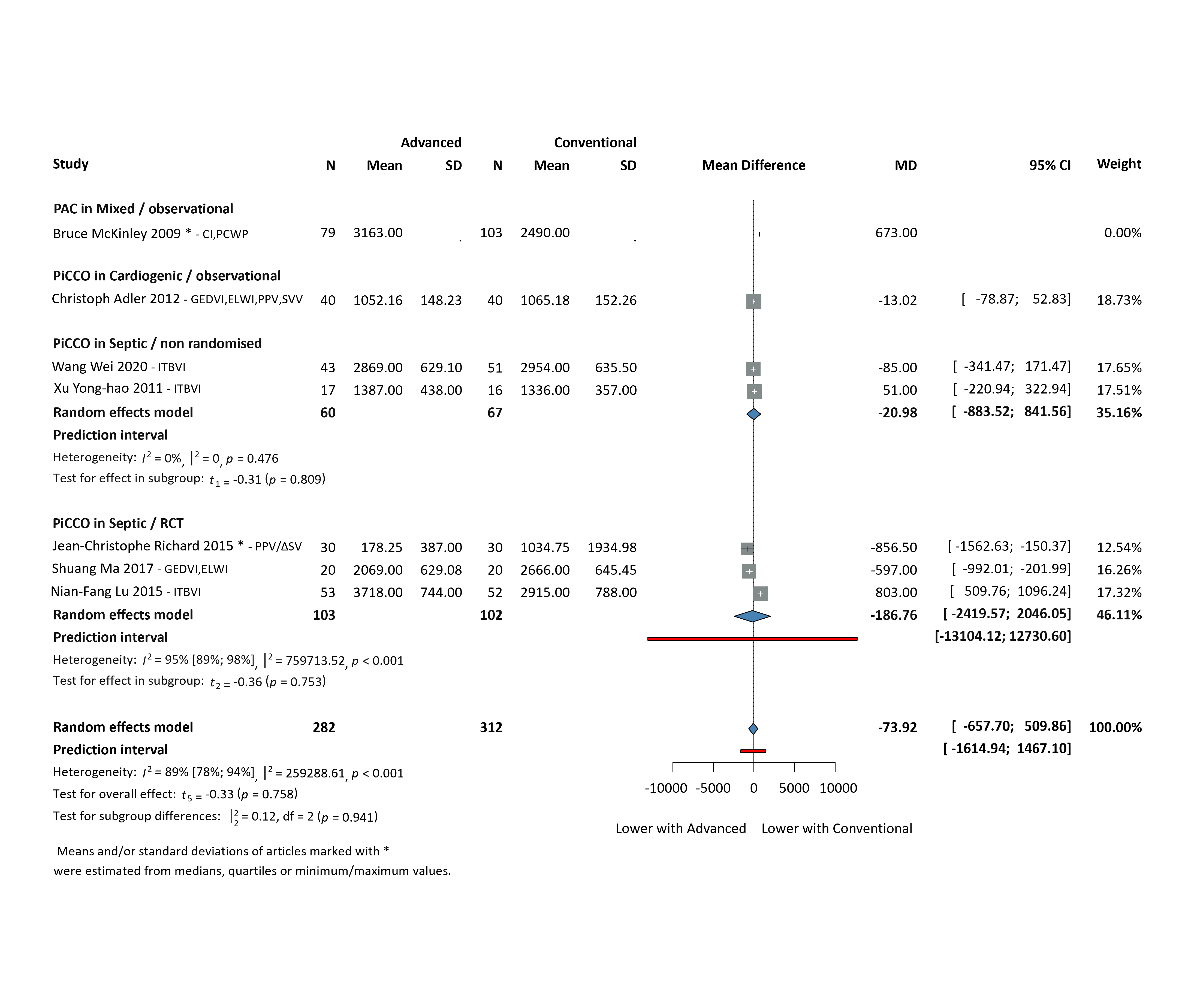  Supplementary Figure 8. Received fluid volumes – 0-6 hours |
| 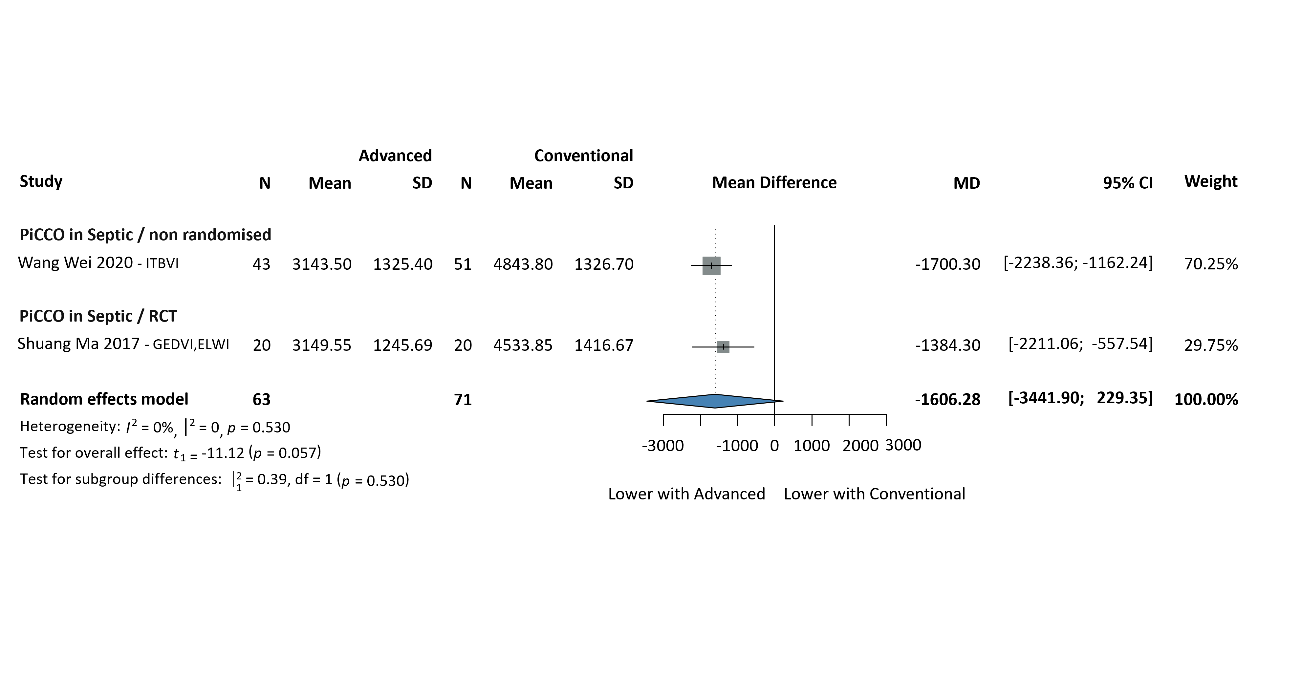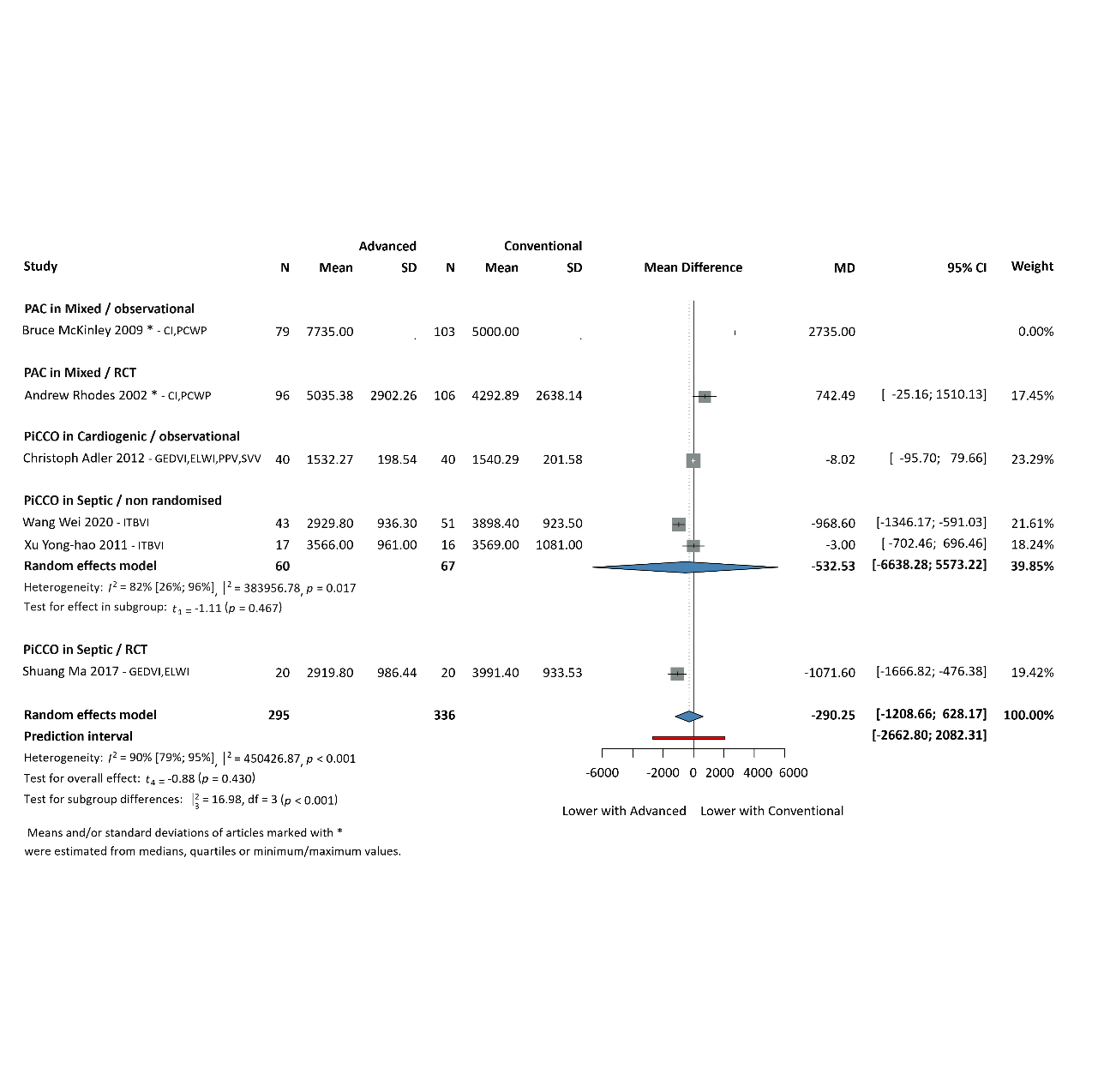  Supplementary Figure 9. Received fluid volumes – 0-24 hours (cumulative)  Supplementary Figure 10. Received fluid volumes – 0-48 hours (cumulative) |
| 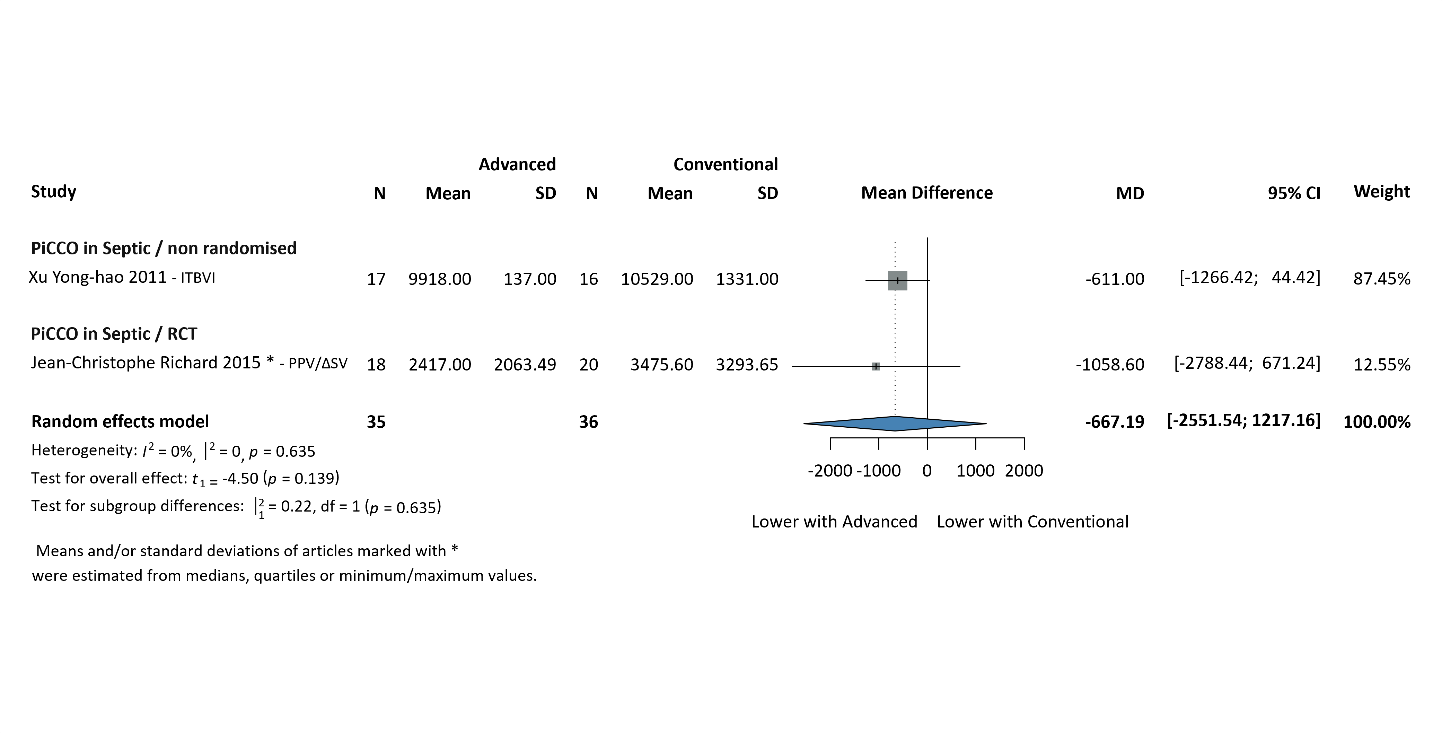  Supplementary Figure 11. Received fluid volumes – 0-72 hours (cumulative) |
|  |
| 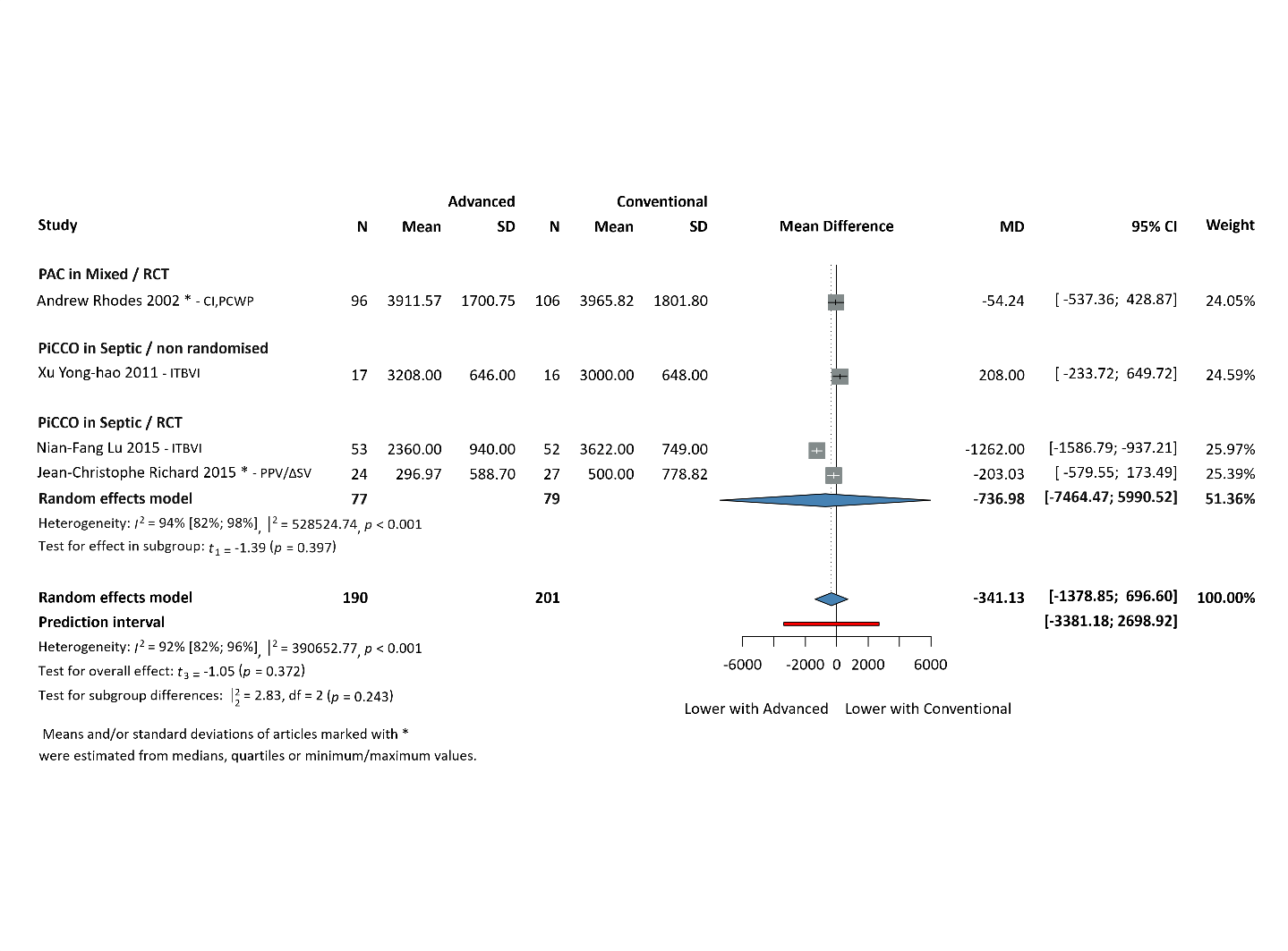  Supplementary Figure 12. Received fluid volumes – 24-48 hours |
| 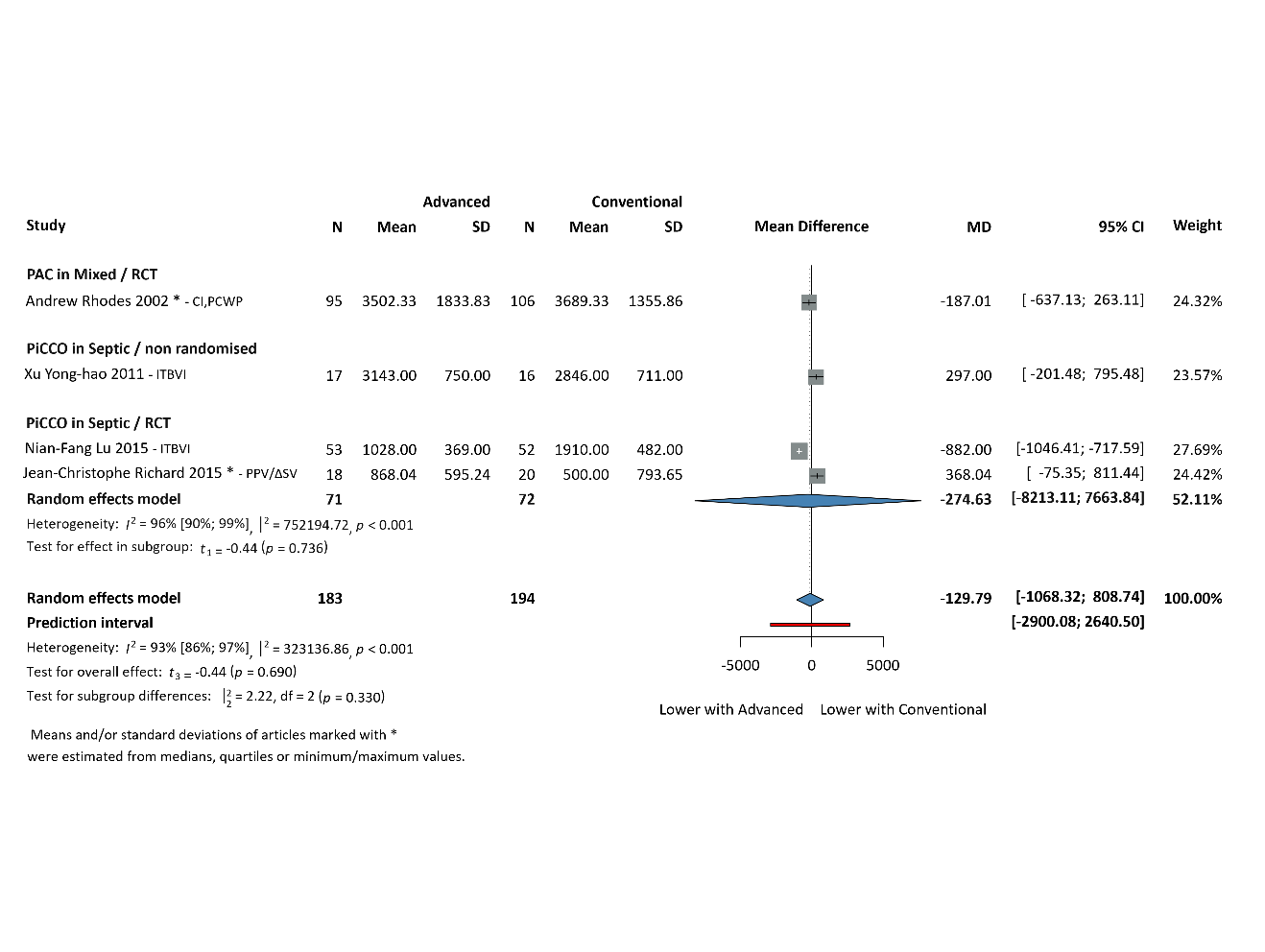  Supplementary Figure 13. Received fluid volumes – 48-72 hours |

# Publication bias figures – Funnel plots

Funnel plots were generated for all analysed outcomes to assess potential publication bias. Egger’s regression test was applied to statistically assess funnel plot asymmetry.

| 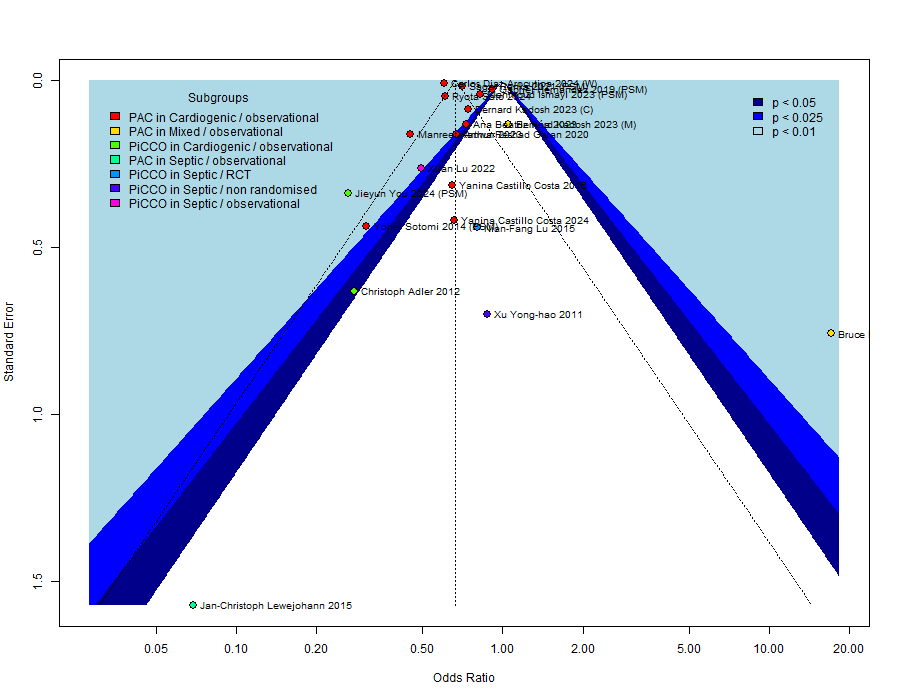 | 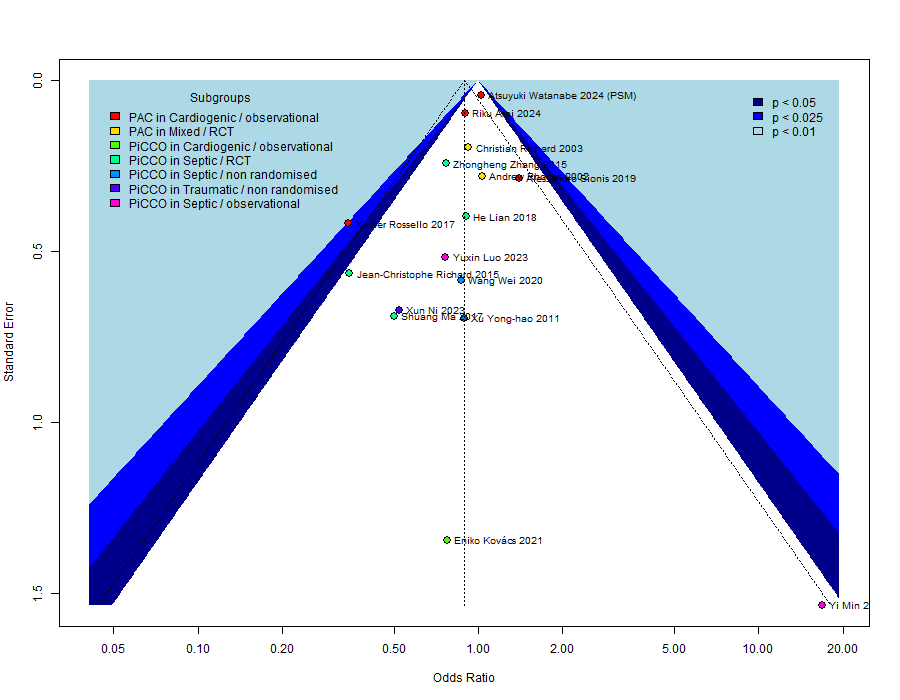 | 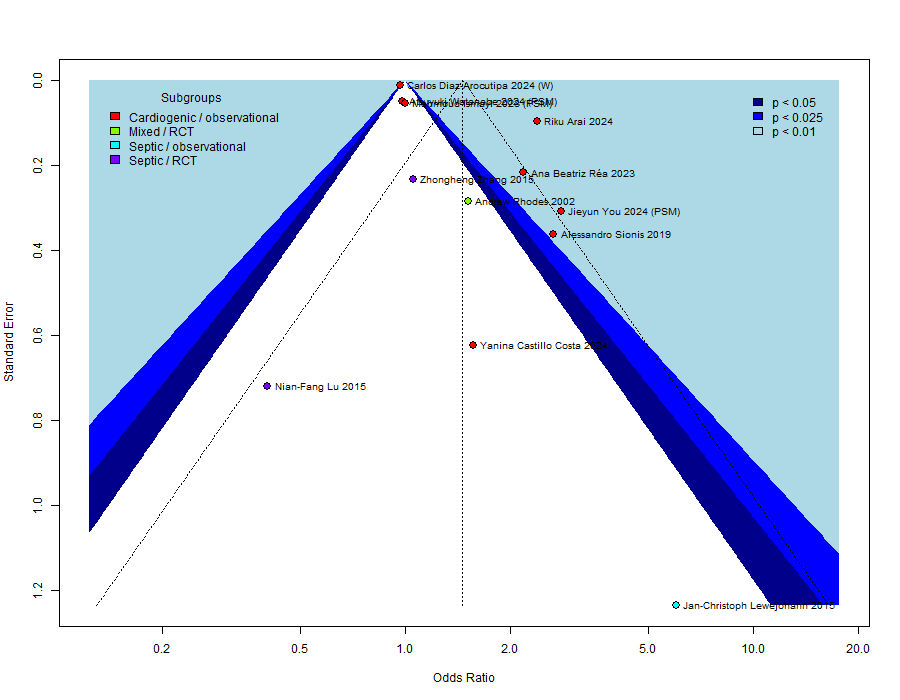 | 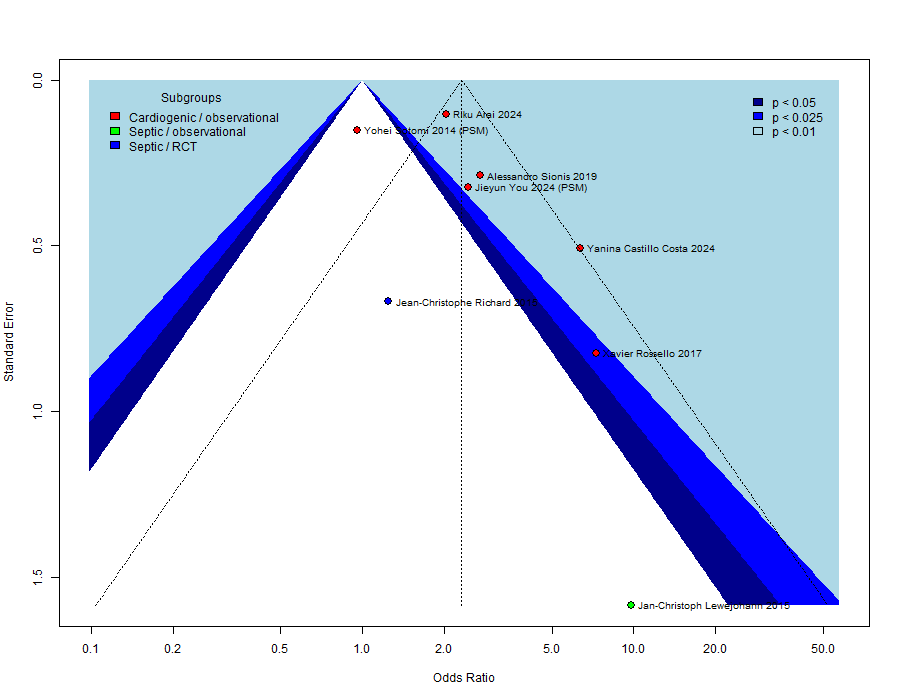 |
| --- | --- | --- | --- |
| *In-hospital mortality* | *30-day mortality* | *Need for vasopressor support* | *Need for inotropic support* |
|  |  |  |  |
| 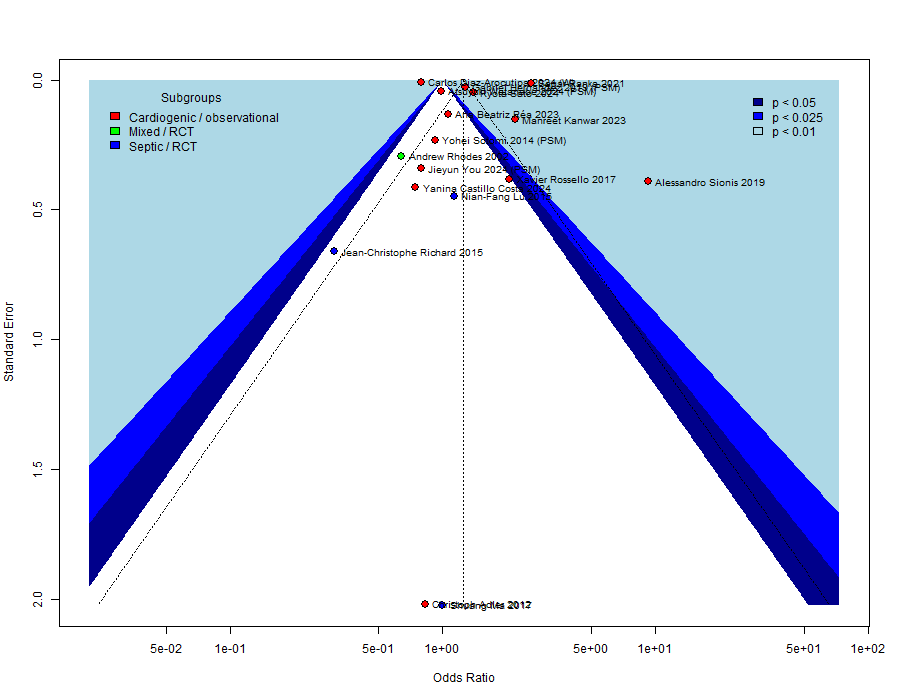 | 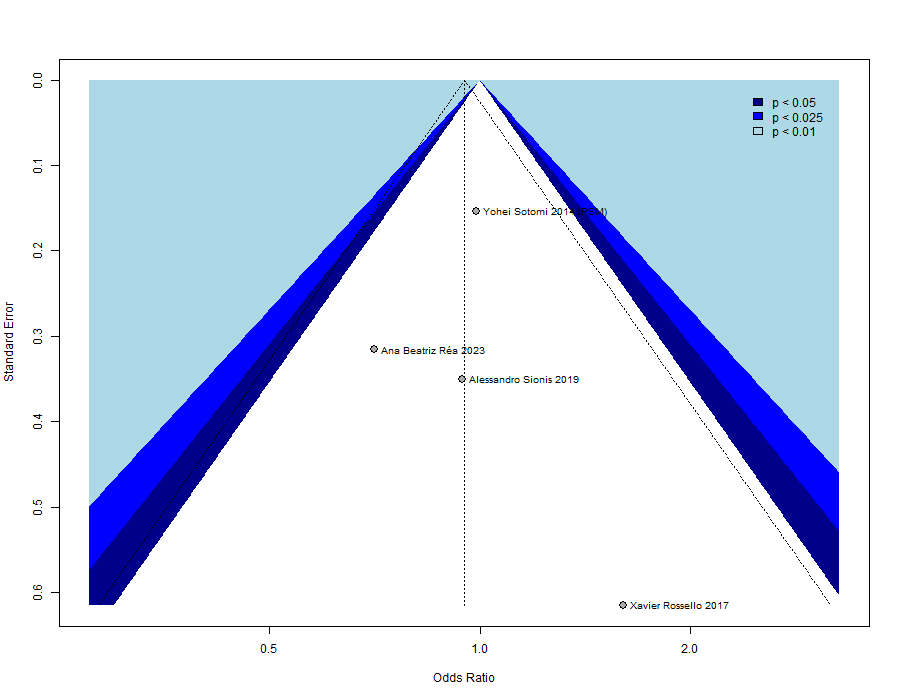 | 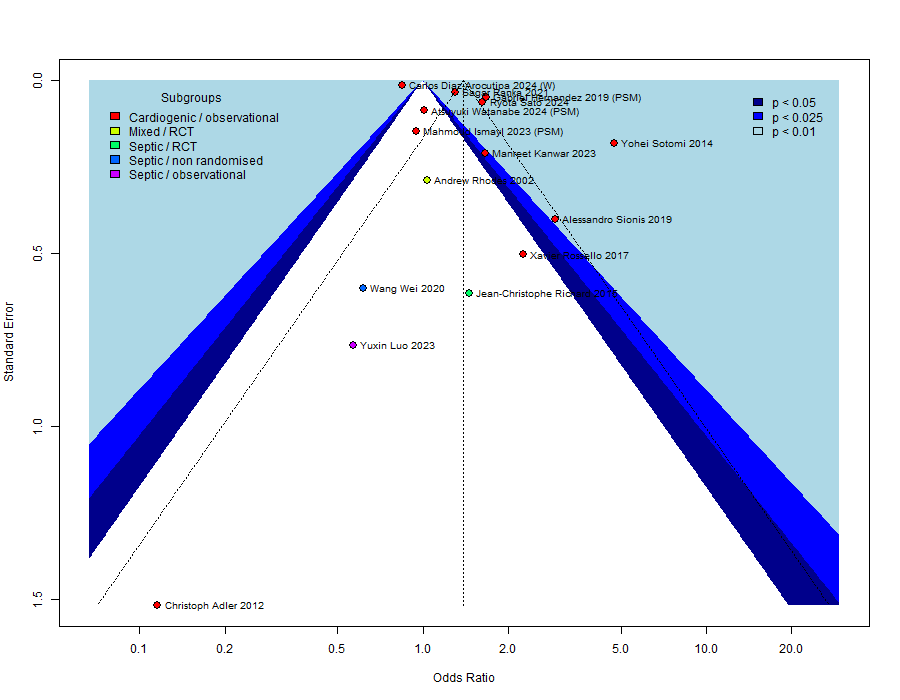 | 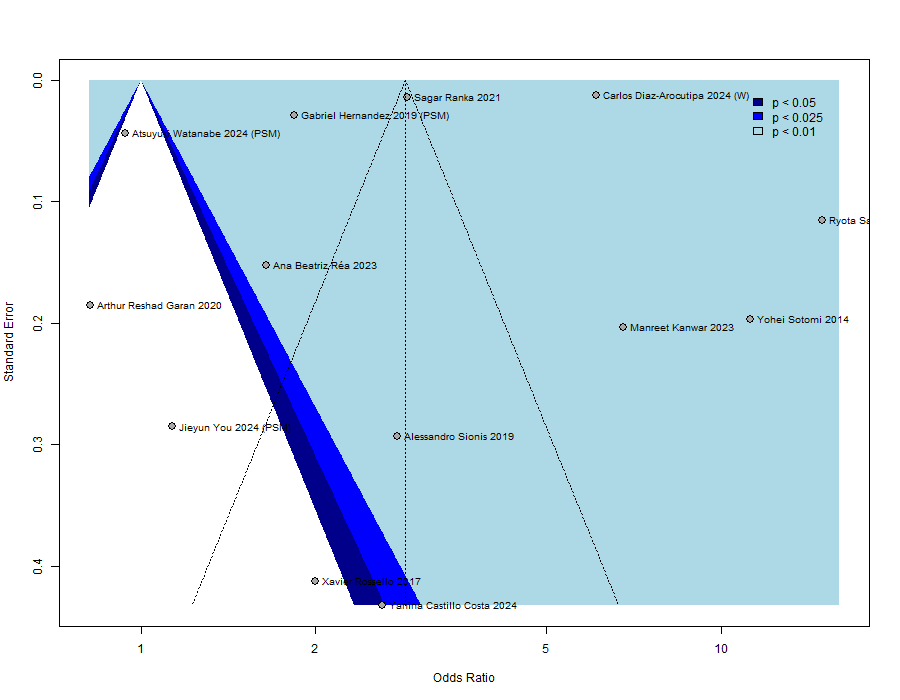 |
| *Need for mechanical ventilation* | *Need for non-invasive ventilation* | *Need for renal replacement therapy* | *Need for mechanical circulatory support* |

| 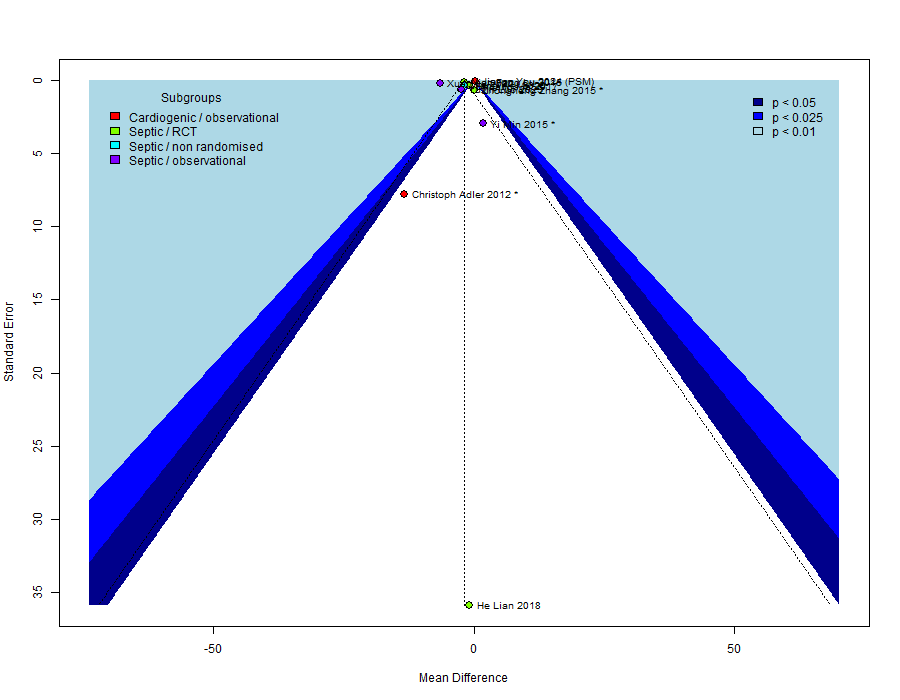 | 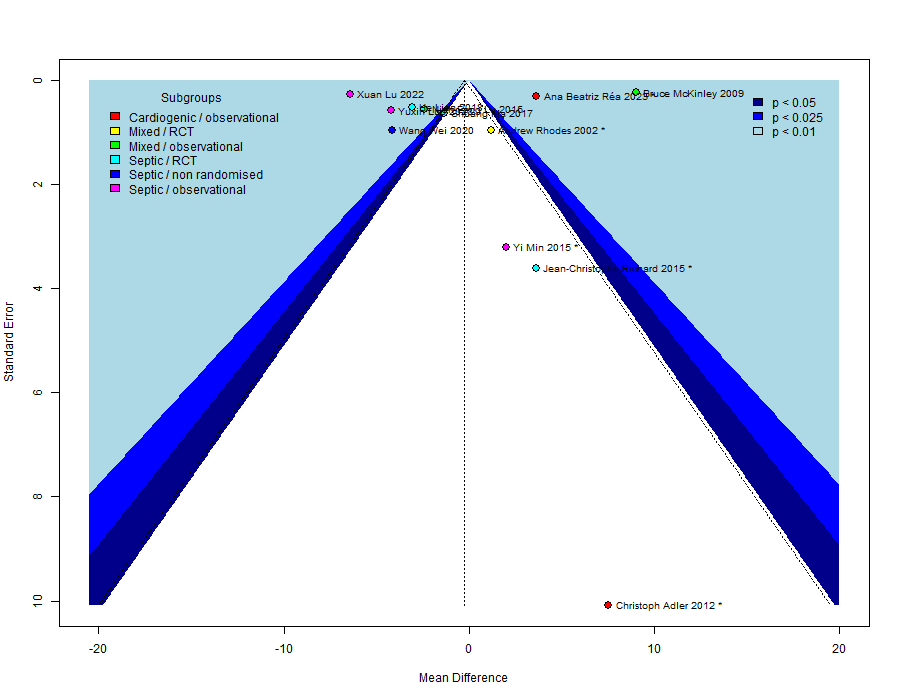 | 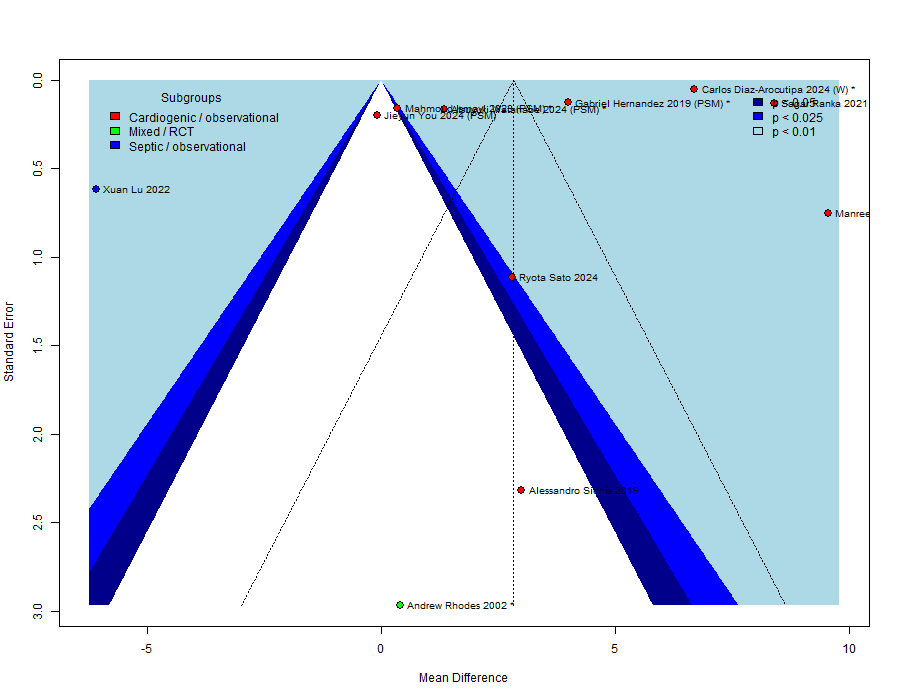 | 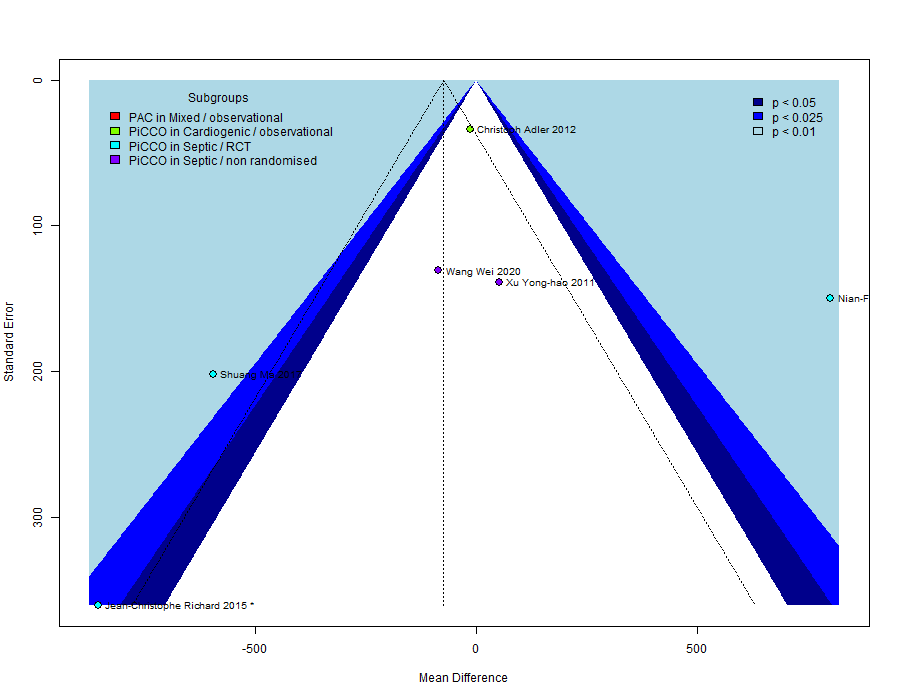 |
| --- | --- | --- | --- |
| *Length of mechanical ventilation* | *Length of ICU stay* | *Length of hospital stay* | *Received fluid volumes / 0-6 h* |
|  |  |  |  |
| 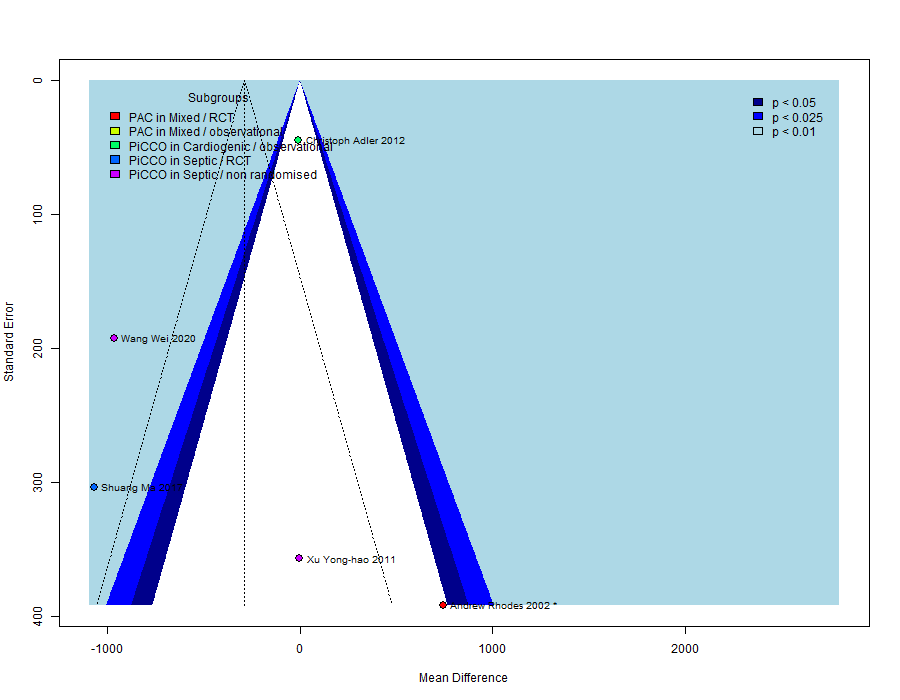 | 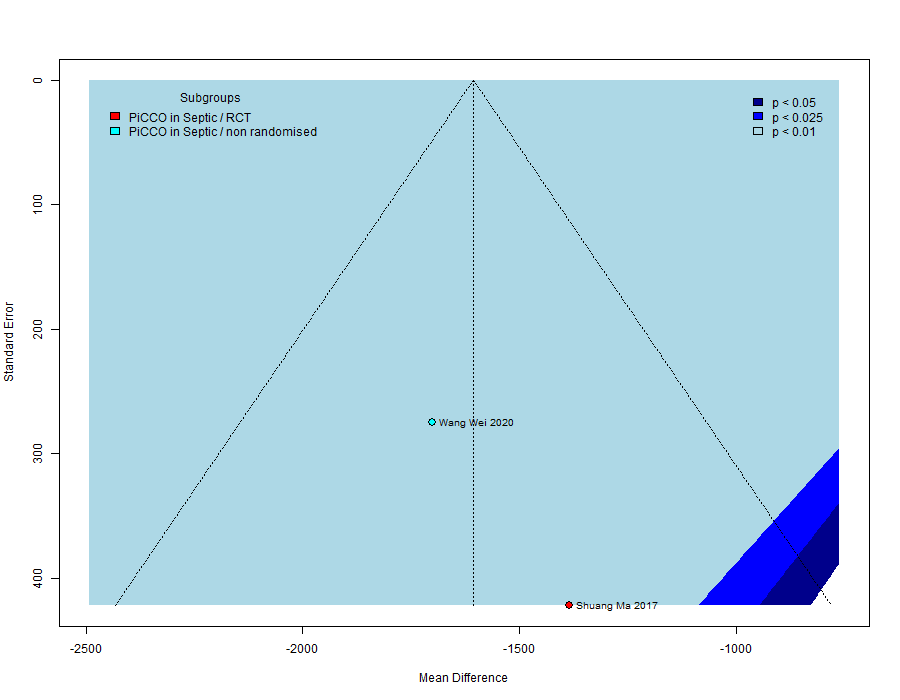 | 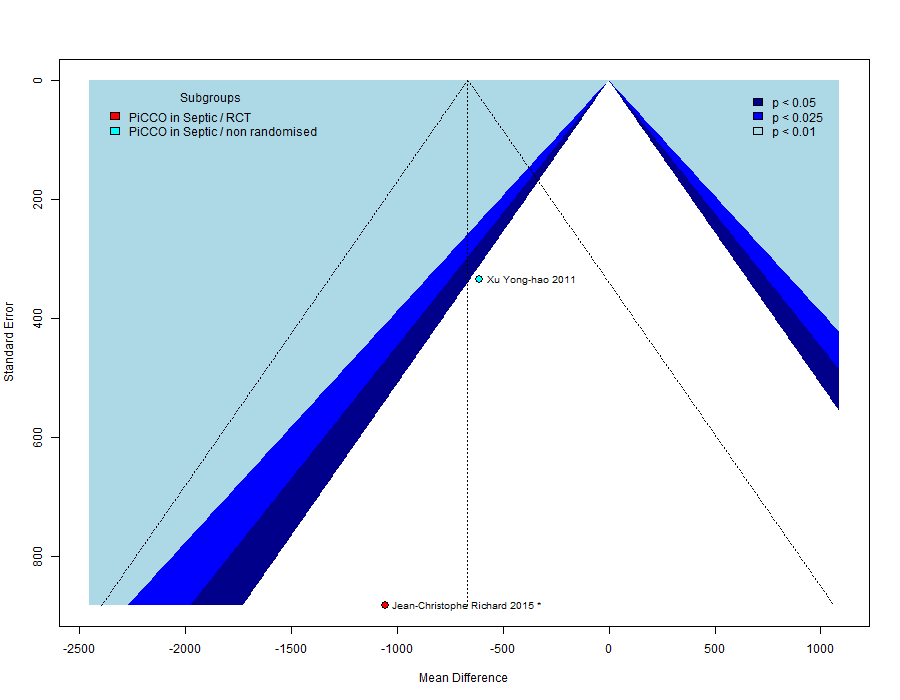 | 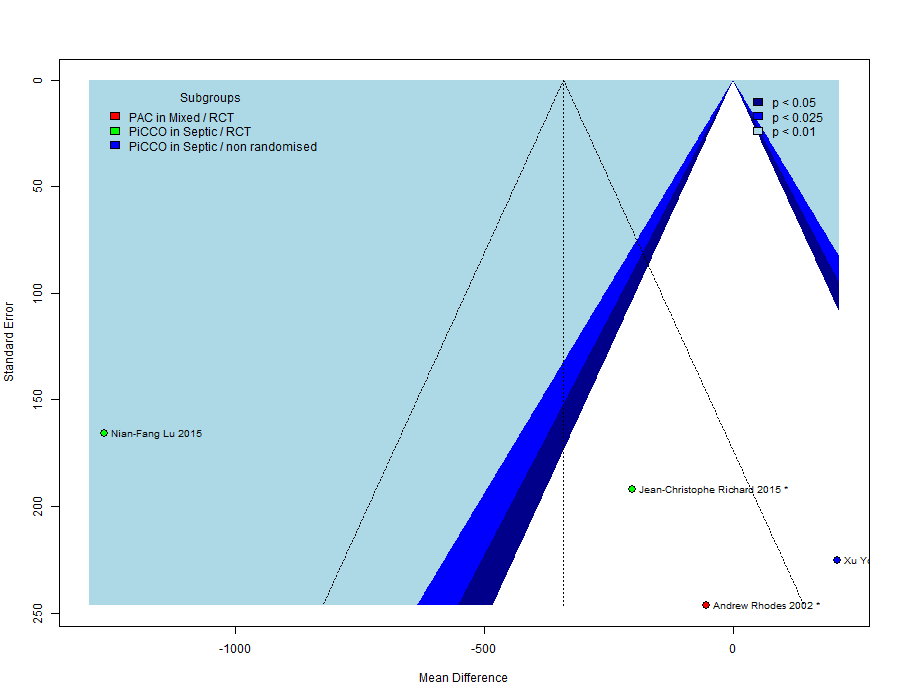 |
| *Received fluid volumes / 0-24 h* | *Received fluid volumes / 0-48 h* | *Received fluid volumes / 0-72 h* | *Received fluid volumes / 24-48 h* |
|  |  |  |  |
| 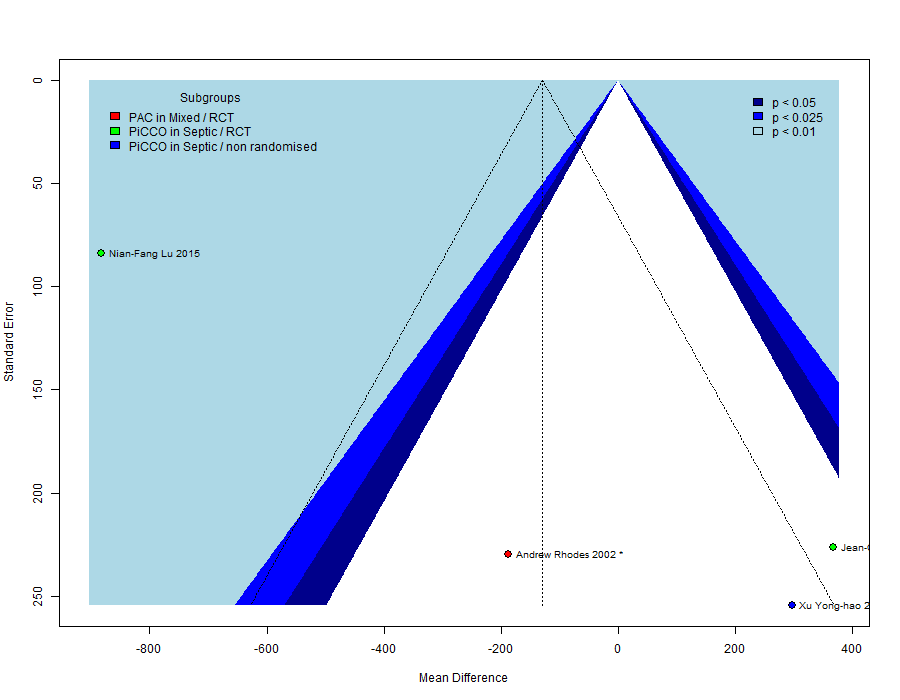 |  |  |  |
| *Received fluid volumes / 48-72 h* |  |  |  |

*Supplementary Figures 13-29. Funnel plots assessing publication bias*

# Risk of bias assessment for each outcome

This table summarises the proportion of included studies judged to be at high risk of bias across the individual domains of the risk of bias assessment tools. For each outcome, we calculated the percentage of studies that were rated as having a high risk of bias in each domain. Randomised controlled trials were assessed using the ROB-2 tool, while non-randomised and observational studies were assessed using the ROBINS-I tool.

|  | **Outcome** | **Domain 1** | **Domain 2** | **Domain 3** | **Domain 4** | **Domain 5** | **Domain 6** | **Domain 7** | **Overall risk of bias** |
| --- | --- | --- | --- | --- | --- | --- | --- | --- | --- |
| RCTs, with the ROB-2 tool | in-hospital mortality  (1 study) | 0% | 0% | 0% | 0% | 0% | - | - | 0% high risk |
|  | 30-day mortality  (6 studies) | 0% | 33.33% | 0% | 16.67% | 0% | - | - | 33.33% high risk |
|  | need for mechanical ventilation (0 studies) | - | - | - | - | - | - | - | - |
|  | need for non-invasive ventilation (0 studies) | - | - | - | - | - | - | - | - |
|  | need for renal replacement therapy  (2 studies) | 0% | 0% | 0% | 0% | 0% | - | - | 0% high risk |
|  | need for inotropic support (1 study) | 0% | 0% | 0% | 0% | 0% | - | - | 0% high risk |
|  | need for vasopressor support (3 studies) | 0% | 0% | 0% | 0% | 0% | - | - | 0% high risk |
|  | need for mechanical circulatory support  (0 studies) | - | - | - | - | - | - | - | - |
|  | length of mechanical ventilation (4 studies) | 0% | 25% | 0% | 0% | 0% | - | - | 25% high risk |
|  | length of ICU stay  (5 studies) | 0% | 20% | 0% | 0% | 0% | - | - | 20% high risk |
|  | length of hospital stay  (1 study) | 0% | 0% | 0% | 0% | 0% | - | - | 0% high risk |
|  | received fluid amounts  (3 studies) | 0% | 0% | 0% | 0% | 0% | - | - | 0% high risk |
| Non-randomised and observational studies with the ROBINS-I tool | in-hospital mortality  (18 studies) | 0% | 100% | 0% | 0% | 11.11% | 0% | 16.67% | 100% high risk |
|  | 30-day mortality  (10 studies) | 20% | 100% | 10% | 10% | 50% | 50% | 40% | 100% high risk |
|  | need for mechanical ventilation (13 studies) | 0% | 100% | 0% | 7.14% | 14.28% | 14.28% | 14.28% | 100% high risk |
|  | need for non-invasive ventilation (4 studies) | 0% | 100% | 0% | 25% | 25% | 50% | 25% | 100% high risk |
|  | need for renal replacement therapy  (13 studies) | 7.69% | 100% | 0% | 7.69% | 7.69% | 23.08% | 15.38% | 100% high risk |
|  | need for inotropic support (7 studies) | 0% | 100% | 0% | 14.28% | 14.28% | 28.57% | 42.86% | 100% high risk |
|  | need for vasopressor support (9 studies) | 0% | 100% | 0% | 0% | 0% | 11.11% | 22.22% | 100% high risk |
|  | need for mechanical circulatory support  (13 studies) | 0% | 100% | 0% | 7.69% | 15.38% | 15.38% | 15.38% | 100% high risk |
|  | length of mechanical ventilation (6 studies) | 33.33% | 100% | 16.67% | 0% | 0% | 16.67% | 50% | 100% high risk |
|  | length of ICU stay  (7 studies) | 28.57% | 100% | 14.28% | 0% | 0% | 14.28% | 28.57% | 100% high risk |
|  | length of hospital stay (10 studies) | 0% | 100% | 0% | 0% | 0% | 10% | 10% | 100% high risk |
|  | received fluid amounts  (4 studies) | 0% | 100% | 0% | 0% | 0% | 0% | 0% | 100% high risk |

*Supplementary Table 5. Proportion of included studies rated at high risk of bias in each domain, by outcome*

# Detailed results of GRADE assessment

The certainty of evidence for each outcome was systematically evaluated using the GRADEpro tool, following the GRADE approach.

| **Certainty assessment** | | | | | | | | | | **№ of patients** | | **Effect** | | | **Certainty** | | **Importance** | |  |  |
| --- | --- | --- | --- | --- | --- | --- | --- | --- | --- | --- | --- | --- | --- | --- | --- | --- | --- | --- | --- | --- |
| **№ of studies** | | **Study design** | | **Risk of bias** | | **Inconsistency** | **Indirectness** | **Imprecision** | **Other considerations** | **therapy guided by advanced hemodynamic monitoring** | **conventional therapy** | **Relative (95% CI)** | **Absolute (95% CI)** | |  |  |  |  |  |  |
| **In-hospital mortality (assessed with: odds ratio)** | | | | | | | | | | | | | | | | | | |  |  |
| 19 | | non-randomised studies | | serious ^a^ | | not serious | not serious | not serious | none | 27359/101355 (27.0%) | 131287/352350 (37.3%) | **OR 0.66** (0.48 to 0.91) | **91 fewer per 1000** (from 151 fewer to 22 fewer) | | ⨁⨁⨁◯ Moderate^a^ | |  | |  |  |
| **30-day mortality (assessed with: odds ratio)** | | | | | | | | | | | | | | | | | | |  |  |
| 16 | | non-randomised studies | | serious ^a^ | | serious | not serious | not serious | none | 2498/6752 (37.0%) | 2330/6184 (37.7%) | **OR 0.89** (0.74 to 1.07) | **27 fewer per 1000** (from 68 fewer to 16 more) | | ⨁⨁◯◯ Low^a^ | |  | |  |  |
| **Need for organ support / inotropic support** | | | | | | | | | | | | | | | | | | |  |  |
| 8 | | non-randomised studies | | serious ^a^ | | not serious | not serious | not serious | strong association | 899/2202 (40.8%) | 480/1599 (30.0%) | **OR 2.32** (1.29 to 4.19) | **199 more per 1000** (from 56 more to 342 more) | | ⨁⨁⨁⨁ High^a^ | |  | |  |  |
| **Need for organ support / vasopressor support** | | | | | | | | | | | | | | | | | | |  |  |
| 12 | | non-randomised studies | | serious ^a^ | | not serious | not serious | not serious | none | 13206/64917 (20.3%) | 50330/261786 (19.2%) | **OR 1.46** (1.05 to 2.04) | **66 more per 1000** (from 8 more to 135 more) | | ⨁⨁⨁◯ Moderate^a^ | |  | |  |  |
| **Need for organ support / mechanical ventilation** | | | | | | | | | | | | | | | | | | |  |  |
| 17 | | non-randomised studies | | serious ^a^ | | not serious | not serious | not serious | none | 44612/99087 (45.0%) | 210849/534291 (39.5%) | **OR 1.25** (0.88 to 1.80) | **54 more per 1000** (from 30 fewer to 145 more) | | ⨁⨁⨁◯ Moderate^a^ | |  | |  |  |
| **Need for organ support / non-invasive ventilation** | | | | | | | | | | | | | | | | | | |  |  |
| 4 | | non-randomised studies | | serious ^a^ | | not serious | not serious | not serious | none | 152/1155 (13.2%) | 168/1240 (13.5%) | **OR 0.95** (0.70 to 1.28) | **6 fewer per 1000** (from 37 fewer to 32 more) | | ⨁⨁⨁◯ Moderate^a^ | |  | |  |  |
| **Need for organ support / renal replacement therapy** | | | | | | | | | | | | | | | | | | |  |  |
| 15 | | non-randomised studies | | serious ^a^ | | not serious | not serious | not serious | none | 8727/103483 (8.4%) | 44048/541770 (8.1%) | **OR 1.39** (1.00 to 1.93) | **28 more per 1000** (from 0 fewer to 65 more) | | ⨁⨁⨁◯ Moderate^a^ | |  | |  |  |
| **Need for organ support / mechanical circulatory support** | | | | | | | | | | | | | | | | | | |  |  |
| 13 | | non-randomised studies | | serious ^a^ | | not serious | not serious | not serious | strong association publication bias suspected ^b^ | 33101/99774 (33.2%) | 87799/537799 (16.3%) | **OR 2.85** (1.62 to 5.02) | **194 more per 1000** (from 77 more to 332 more) | | ⨁⨁⨁◯ Moderate^a,b^ | |  | |  |  |
| **Length of stay / ICU-stay** | | | | | | | | | | | | | | | | | | |  |  |
| 12 | | non-randomised studies | | serious ^a^ | | not serious | not serious | not serious | none | 1118 | 1183 | - | MD **0.26 days lower** (3.24 lower to 2.72 higher) | | ⨁⨁⨁◯ Moderate^a^ | |  | |  |  |
| **Length of stay / hospital-stay** | | | | | | | | | | | | | | | | | | |  |  |
| 11 | | non-randomised studies | | serious ^a^ | | not serious | not serious | not serious | strong association publication bias suspected ^b^ | 102751 | 537888 | - | MD **2.82 days higher** (0.2 lower to 5.84 higher) | | ⨁⨁⨁◯ Moderate^a,b^ | |  | |  |  |
| **Length of organ support / mechanical ventilation** | | | | | | | | | | | | | | | | | | |  |  |
| 10 | | non-randomised studies | | serious ^a^ | | not serious | not serious | not serious | none | 731 | 762 | - | MD **1.81 days lower** (3.72 lower to 0.09 higher) | | ⨁⨁⨁◯ Moderate^a^ | |  | |  |  |
| **Received fluid volumes / 0-6 hours** | | | | | | | | | | | | | | | | | | |  |  |
| 7 | | non-randomised studies | | serious ^a^ | | not serious | not serious | not serious | none | 282 | 312 | - | MD **73.92 ml lower** (657.7 lower to 509.86 higher) | | ⨁⨁⨁◯ Moderate^a^ | |  | |  |  |
| **Received fluid volumes / 24-48 hours** | | | | | | | | | | | | | | | | | | |  |  |
| 4 | | non-randomised studies | | serious ^a^ | | not serious | not serious | not serious | none | 190 | 201 | - | MD **341.18 ml lower** (1378.85 lower to 696.6 higher) | | ⨁⨁⨁◯ Moderate^a^ | |  | |  |  |
| **Received fluid volumes / 48-72 hours** | | | | | | | | | | | | | | | | | | |  |  |
| 4 | | non-randomised studies | | serious ^a^ | | not serious | not serious | not serious | none | 183 | 194 | - | MD **129.79 ml lower** (1068.32 lower to 808.74 higher) | | ⨁⨁⨁◯ Moderate^a^ | |  | |  |  |
| **Received fluid volumes / 0-24 hours (cumulative)** | | | | | | | | | | | | | | | | | | |  |  |
| 6 | | non-randomised studies | | serious ^a^ | | not serious | not serious | not serious | none | 295 | 336 | - | MD **290.25 ml lower** (1208.66 lower to 628.17 higher) | | ⨁⨁⨁◯ Moderate^a^ | |  | |  |  |
| **Received fluid volumes / 0-48 hours (cumulative)** | | | | | | | | | | | | | | | | | | | | |
| 2 | | non-randomised studies | | serious ^a^ | | not serious | | not serious | not serious | none | 63 | 71 | - | | MD **1606.28 ml lower** (3441.9 lower to 229.35 higher) | | ⨁⨁⨁◯ Moderate^a^ | |  | |
| **Received fluid volumes / 0-72 hours (cumulative)** | | | | | | | | | | | | | | | | | | | | |
| 2 | | non-randomised studies | | serious ^a^ | | not serious | | not serious | not serious | none | 35 | 36 | - | | MD **667.19 ml lower** (2551.54 lower to 1217.16 higher) | | ⨁⨁⨁◯ Moderate^a^ | |  | |

*Supplementary Table 6. Certainty of evidence assessment for each outcome
CI: confidence interval; MD: mean difference; OR: odds ratio****Explanations:*** *a. Serious risk of bias due to classification of interventions (ROBINS-I tool, Domain 2) in observational studies.
b. Funnel plot asymmetry suggested potential publication bias, though this likely reflects differences in study design and sample size imbalance*

# PRISMA reporting guidelines

| **Section and Topic** | **Item #** | **Checklist item** |
| --- | --- | --- |
| **TITLE** | | |
| Title | 1 | Identify the report as a systematic review. |
| **ABSTRACT** | | |
| Abstract | 2 | See the PRISMA 2020 for Abstracts checklist. |
| **INTRODUCTION** | | |
| Rationale | 3 | Describe the rationale for the review in the context of existing knowledge. |
| Objectives | 4 | Provide an explicit statement of the objective(s) or question(s) the review addresses. |
| **METHODS** | | |
| Eligibility criteria | 5 | Specify the inclusion and exclusion criteria for the review and how studies were grouped for the syntheses. |
| Information sources | 6 | Specify all databases, registers, websites, organisations, reference lists and other sources searched or consulted to identify studies. Specify the date when each source was last searched or consulted. |
| Search strategy | 7 | Present the full search strategies for all databases, registers and websites, including any filters and limits used. |
| Selection process | 8 | Specify the methods used to decide whether a study met the inclusion criteria of the review, including how many reviewers screened each record and each report retrieved, whether they worked independently, and if applicable, details of automation tools used in the process. |
| Data collection process | 9 | Specify the methods used to collect data from reports, including how many reviewers collected data from each report, whether they worked independently, any processes for obtaining or confirming data from study investigators, and if applicable, details of automation tools used in the process. |
| Data items | 10a | List and define all outcomes for which data were sought. Specify whether all results that were compatible with each outcome domain in each study were sought (e.g. for all measures, time points, analyses), and if not, the methods used to decide which results to collect. |
|  | 10b | List and define all other variables for which data were sought (e.g. participant and intervention characteristics, funding sources). Describe any assumptions made about any missing or unclear information. |
| Study risk of bias assessment | 11 | Specify the methods used to assess risk of bias in the included studies, including details of the tool(s) used, how many reviewers assessed each study and whether they worked independently, and if applicable, details of automation tools used in the process. |
| Effect measures | 12 | Specify for each outcome the effect measure(s) (e.g. risk ratio, mean difference) used in the synthesis or presentation of results. |
| Synthesis methods | 13a | Describe the processes used to decide which studies were eligible for each synthesis (e.g. tabulating the study intervention characteristics and comparing against the planned groups for each synthesis (item #5)). |
|  | 13b | Describe any methods required to prepare the data for presentation or synthesis, such as handling of missing summary statistics, or data conversions. |
|  | 13c | Describe any methods used to tabulate or visually display results of individual studies and syntheses. |
|  | 13d | Describe any methods used to synthesize results and provide a rationale for the choice(s). If meta-analysis was performed, describe the model(s), method(s) to identify the presence and extent of statistical heterogeneity, and software package(s) used. |
|  | 13e | Describe any methods used to explore possible causes of heterogeneity among study results (e.g. subgroup analysis, meta-regression). |
|  | 13f | Describe any sensitivity analyses conducted to assess robustness of the synthesized results. |
| Reporting bias assessment | 14 | Describe any methods used to assess risk of bias due to missing results in a synthesis (arising from reporting biases). |
| Certainty assessment | 15 | Describe any methods used to assess certainty (or confidence) in the body of evidence for an outcome. |
| **RESULTS** | | |
| Study selection | 16a | Describe the results of the search and selection process, from the number of records identified in the search to the number of studies included in the review, ideally using a flow diagram. |
|  | 16b | Cite studies that might appear to meet the inclusion criteria, but which were excluded, and explain why they were excluded. |
| Study characteristics | 17 | Cite each included study and present its characteristics. |
| Risk of bias in studies | 18 | Present assessments of risk of bias for each included study. |
| Results of individual studies | 19 | For all outcomes, present, for each study: (a) summary statistics for each group (where appropriate) and (b) an effect estimate and its precision (e.g. confidence/credible interval), ideally using structured tables or plots. |
| Results of syntheses | 20a | For each synthesis, briefly summarise the characteristics and risk of bias among contributing studies. |
|  | 20b | Present results of all statistical syntheses conducted. If meta-analysis was done, present for each the summary estimate and its precision (e.g. confidence/credible interval) and measures of statistical heterogeneity. If comparing groups, describe the direction of the effect. |
|  | 20c | Present results of all investigations of possible causes of heterogeneity among study results. |
|  | 20d | Present results of all sensitivity analyses conducted to assess the robustness of the synthesized results. |
| Reporting biases | 21 | Present assessments of risk of bias due to missing results (arising from reporting biases) for each synthesis assessed. |
| Certainty of evidence | 22 | Present assessments of certainty (or confidence) in the body of evidence for each outcome assessed. |
| **DISCUSSION** | | |
| Discussion | 23a | Provide a general interpretation of the results in the context of other evidence. |
|  | 23b | Discuss any limitations of the evidence included in the review. |
|  | 23c | Discuss any limitations of the review processes used. |
|  | 23d | Discuss implications of the results for practice, policy, and future research. |
| **OTHER INFORMATION** | | |
| Registration and protocol | 24a | Provide registration information for the review, including register name and registration number, or state that the review was not registered. |
|  | 24b | Indicate where the review protocol can be accessed, or state that a protocol was not prepared. |
|  | 24c | Describe and explain any amendments to information provided at registration or in the protocol. |
| Support | 25 | Describe sources of financial or non-financial support for the review, and the role of the funders or sponsors in the review. |
| Competing interests | 26 | Declare any competing interests of review authors. |
| Availability of data, code and other materials | 27 | Report which of the following are publicly available and where they can be found: template data collection forms; data extracted from included studies; data used for all analyses; analytic code; any other materials used in the review. |
